# Exploring the promoter regions of cancer predisposition genes in patients with triple-negative breast cancer reveals the presence of rare germline variants

**DOI:** 10.1093/oncolo/oyaf052

**Published:** 2025-05-08

**Authors:** Michela Palleschi, Alessandra Virga, Emanuela Scarpi, Eugenio Fonzi, Antonino Musolino, Filippo Merloni, Samanta Sarti, Rita Danesi, Mila Ravegnani, Chiara Casadei, Marianna Sirico, Caterina Gianni, Roberta Maltoni, Sara Bravaccini, Daniele Calistri, Valentina Arcangeli, Valentina Zampiga, Ilaria Cangini, Erika Bandini, Francesca Mannozzi, Fabio Falcini, Giovanni Martinelli, Ugo De Giorgi, Paola Ulivi, Gianluca Tedaldi

**Affiliations:** Medical Oncology, Breast & GYN Unit, IRCCS Istituto Romagnolo per lo Studio dei Tumori (IRST) “Dino Amadori”, Meldola, 47014, Italy; Biosciences Laboratory, IRCCS Istituto Romagnolo per lo Studio dei Tumori (IRST) “Dino Amadori”, Meldola, 47014, Italy; Unit of Biostatistics and Clinical Trials, IRCCS Istituto Romagnolo per lo Studio dei Tumori (IRST) “Dino Amadori”, 47014, Meldola, Italy; Unit of Biostatistics and Clinical Trials, IRCCS Istituto Romagnolo per lo Studio dei Tumori (IRST) “Dino Amadori”, 47014, Meldola, Italy; Medical Oncology, Breast & GYN Unit, IRCCS Istituto Romagnolo per lo Studio dei Tumori (IRST) “Dino Amadori”, Meldola, 47014, Italy; Department of Medical and Surgical Science, University of Bologna, Bologna, 40138, Italy; Medical Oncology, Breast & GYN Unit, IRCCS Istituto Romagnolo per lo Studio dei Tumori (IRST) “Dino Amadori”, Meldola, 47014, Italy; Medical Oncology, Breast & GYN Unit, IRCCS Istituto Romagnolo per lo Studio dei Tumori (IRST) “Dino Amadori”, Meldola, 47014, Italy; Emilia-Romagna Cancer Registry, Romagna Unit, IRCCS Istituto Romagnolo per lo Studio dei Tumori (IRST) "Dino Amadori", Meldola, 47014, Italy; Emilia-Romagna Cancer Registry, Romagna Unit, IRCCS Istituto Romagnolo per lo Studio dei Tumori (IRST) "Dino Amadori", Meldola, 47014, Italy; Medical Oncology, Breast & GYN Unit, IRCCS Istituto Romagnolo per lo Studio dei Tumori (IRST) “Dino Amadori”, Meldola, 47014, Italy; Medical Oncology, Breast & GYN Unit, IRCCS Istituto Romagnolo per lo Studio dei Tumori (IRST) “Dino Amadori”, Meldola, 47014, Italy; Medical Oncology, Breast & GYN Unit, IRCCS Istituto Romagnolo per lo Studio dei Tumori (IRST) “Dino Amadori”, Meldola, 47014, Italy; Medical Oncology, Breast & GYN Unit, IRCCS Istituto Romagnolo per lo Studio dei Tumori (IRST) “Dino Amadori”, Meldola, 47014, Italy; Biosciences Laboratory, IRCCS Istituto Romagnolo per lo Studio dei Tumori (IRST) “Dino Amadori”, Meldola, 47014, Italy; Biosciences Laboratory, IRCCS Istituto Romagnolo per lo Studio dei Tumori (IRST) “Dino Amadori”, Meldola, 47014, Italy; Emilia-Romagna Cancer Registry, Romagna Unit, IRCCS Istituto Romagnolo per lo Studio dei Tumori (IRST) "Dino Amadori", Meldola, 47014, Italy; Biosciences Laboratory, IRCCS Istituto Romagnolo per lo Studio dei Tumori (IRST) “Dino Amadori”, Meldola, 47014, Italy; Biosciences Laboratory, IRCCS Istituto Romagnolo per lo Studio dei Tumori (IRST) “Dino Amadori”, Meldola, 47014, Italy; Biosciences Laboratory, IRCCS Istituto Romagnolo per lo Studio dei Tumori (IRST) “Dino Amadori”, Meldola, 47014, Italy; Unit of Biostatistics and Clinical Trials, IRCCS Istituto Romagnolo per lo Studio dei Tumori (IRST) “Dino Amadori”, 47014, Meldola, Italy; Emilia-Romagna Cancer Registry, Romagna Unit, IRCCS Istituto Romagnolo per lo Studio dei Tumori (IRST) "Dino Amadori", Meldola, 47014, Italy; Local Health Authority, Cancer Prevention Unit, Forlì, 47121, Italy; Department of Hematology and Sciences Oncology, Institute of Haematology “L. and A. Seràgnoli”, S. Orsola University Hospital, Bologna, 40138, Italy; Department of Medical Oncology, IRCCS Istituto Romagnolo per lo Studio dei Tumori (IRST) “Dino Amadori”, Meldola, 47014, Italy; Biosciences Laboratory, IRCCS Istituto Romagnolo per lo Studio dei Tumori (IRST) “Dino Amadori”, Meldola, 47014, Italy; Biosciences Laboratory, IRCCS Istituto Romagnolo per lo Studio dei Tumori (IRST) “Dino Amadori”, Meldola, 47014, Italy

**Keywords:** hereditary breast cancer, triple-negative breast cancer, promoter variants, regulatory regions, cancer predisposition

## Abstract

**Background:**

Current genetic screening for predisposition to breast cancer (BC) is limited to *BRCA1/2* exons and intron/exon boundaries, and limited information exists about the impact of variants in *BRCA1/2* non-coding regions. The majority of alterations identified in these regions remain unclassified, but evidence of the impact of variants in the regulatory regions on cancer risk and response to treatment is emerging.

**Patients and methods:**

This project aimed to investigate the prevalence of germline variants in the non-coding regulatory regions of *BRCA1/2* and other BC predisposition genes in patients with triple-negative BC (TNBC) selected for age at cancer diagnosis and/or family history of cancer. The study also aims to investigate the relationship between these variants and clinical outcomes such as overall survival, disease-free survival (DFS), and response to treatment. We analyzed a Next-Generation Sequencing (NGS) custom panel of promoter regions of 28 genes involved in BC predisposition on 144 patients with TNBC previously tested wild type for coding regions of *BRCA1/2*.

**Results:**

The NGS analysis identified 635 rare variants in promoter regions of the 28 genes. Among the 144 patients, for 75 with available clinical data, rare germline variants in *BRCA2* promoter were statistically significantly related to worse overall survival (OS) (*P*-value = .017). No differences in DFS and OS were found for the other genes. Rare variants in the *CDH1* promoter were related to the highest percentage of non-pathological complete response after neoadjuvant chemotherapy (*P* = .0273); *MLH1* and *PALB2* rare non-coding variants were found to be both related to bilateral BC (*P* = .0146 and *P* = .0005, respectively) and *ATM* promoter variants were associated with a positive family history (*P* = .041).

**Conclusion:**

Our results underscore the importance of searching for rare germline variants in regulatory regions of cancer predisposition genes in patients with TNBC, since these variants can be associated with an increased cancer risk.

**Implications for practice**

Current genetic screening for inherited diseases is mainly focused on exonic regions, in particular the available commercial tests cover principally the coding sequence or sometimes only hotspot position in several genes. The clinical importance of studying germline variants in the promoter regions of *BRCA1/2* and other predisposition genes in patients with TNBC is critical for risk stratification and optimizing treatment response in hereditary cancer. Current genetic screening of noncoding regions faces limitations in understanding the effective role of molecular alterations for patients with inherited syndromes. Our study examined the regulatory and promoter regions of 28 cancer predisposition genes to highlight the potential correlation between gene variants and clinical outcomes. We identified several variants in the promoter regions of cancer predisposition genes that could increase breast cancer risk. The results suggested a significant association between *BRCA2* variants and worse OS, while *CDH1, MLH1, PALB2* and *ATM* variants showed relationships with treatment response, disease characteristics and family history, respectively. However, more research is needed into the role of genetic alterations in the regulatory regions of cancer susceptibility genes.

## Introduction

Triple-negative breast cancer (TNBC) is a type of breast cancer (BC) in which cells do not have estrogen or progesterone receptors (ER or PR) and do not contain high levels of HER2 receptor. TNBC accounts for about 10%-15% of all BCs.^1^ Women under 40 years, who are Black, or who have a *BRCA1/2* pathogenic variant are more likely to develop TNBC.^2^ More than 75% of BCs that develop in carriers of a *BRCA1/2* mutation (*BRCAmut*) are TNBC. TNBC is usually more aggressive, harder to treat, and more likely to recur than cancers that are hormone receptor-positive.

The presence of *BRCA1/2* variants can affect the treatment of TNBC in several ways:

- PARP inhibitors (PARPi): women with a *BRCAmut* TNBC may benefit from targeted therapies such as PARPi. PARPi are drugs that block an enzyme called PARP, which helps repair damaged DNA. In women with a *BRCAmut* TNBC, PARPi can be effective because they target cells that are already deficient in DNA repair mechanisms. Several studies have shown that PARPi can improve survival in women with *BRCAmut* TNBC.^3^
- Chemotherapy: standard neoadjuvant chemotherapy remains the standard of care for early-stage TNBC, regardless of *BRCA1/2* status. However, some studies have suggested that women with *BRCAmut* TNBC may respond differently to chemotherapy than non-*BRCAmut* TNBC. For example, a systematic review and meta-analysis found that *BRCAmut* TNBC had a higher response rate to platinum-based chemotherapy than non-*BRCAmut* TNBC.^4^
- Clinical trials: women with *BRCAmut* TNBC may be eligible for clinical trials of new treatments. For example, a recent study found that the combination of a PARPi and immunotherapy (pembrolizumab) improved progression-free survival in women with *BRCAmut* TNBC.^5^

**Figure 1.**
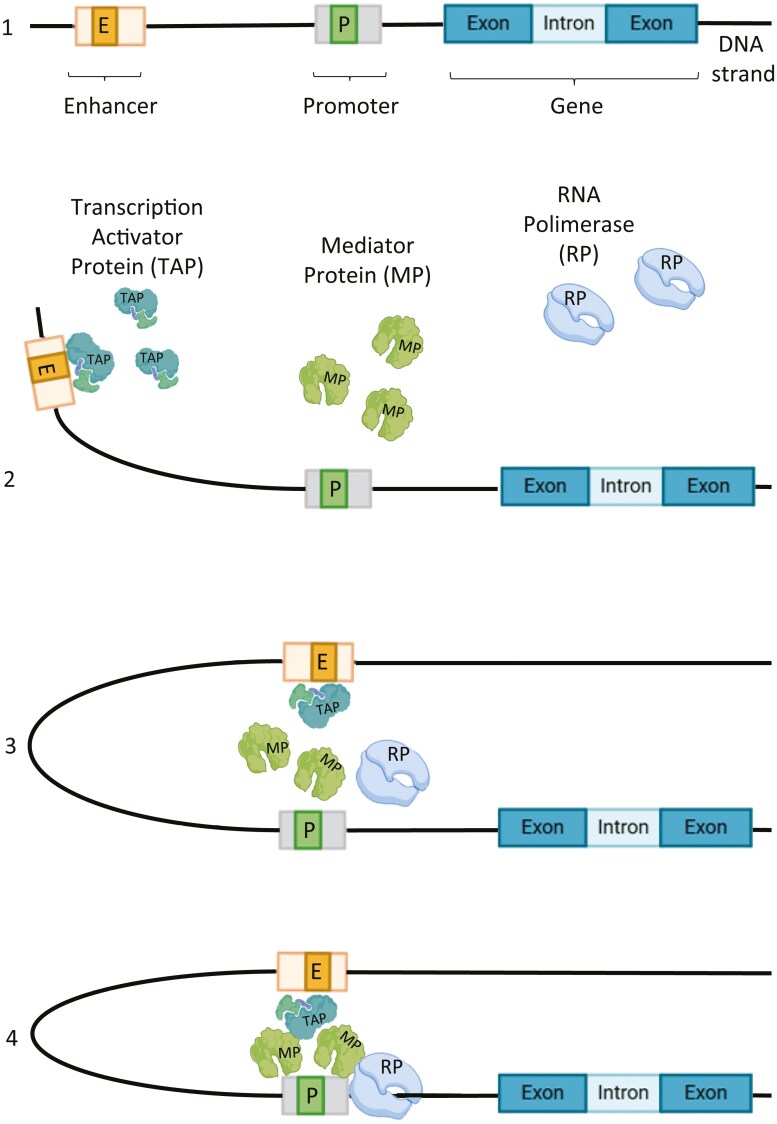
The explanatory figure illustrating the role and structure of enhancers, promoters, and regulatory regions in human genes.

Current genetic screening for *BRCA1/2* variants is limited to the coding exons and intron/exon boundaries of *BRCA1* and *BRCA2* genes. However, thanks to the wide use of Next-Generation Sequencing (NGS), the number of genes and genomic alterations suspected to be involved in cancer predisposition has dramatically increased.^6,7^ However, evidence of non-coding variants’ impact on cancer risk and response to treatment begins to emerge.^8^ Despite this, limited information currently exists about the impact of variants in promoter regions, and the majority of variants that were identified in these regions remain unclassified. Recent studies have demonstrated that alterations in these regions, may alter the transcriptional activities of *BRCA1* and *BRCA2*, potentially leading to an increased susceptibility to breast and ovarian cancers (BOCs), as well as other malignancies such as pancreatic and prostate cancers.^9,10^ Promoter and regulatory regions are essential components of the genome that control gene expression by facilitating the binding of transcription factors and RNA polymerase, thereby influencing cellular functions and developmental processes (**Figure 1**). These regions can modulate the timing, location, and level of gene expression, which is crucial for maintaining normal physiological functions and responding to environmental changes.^11^ Understanding the dynamics of these regulatory elements is vital for elucidating the complex mechanisms underlying gene regulation and its implications for health and disease.^12^

Our study aims to investigate the prevalence of rare germline variants in the regulatory regions of BC predisposition genes in patients with risk factors: TNBC, early onset (< 35 years) and/or family history of BOCs, and/or bilateral BC. The study also aims to investigate the relationship between these variants and clinical outcomes such as overall survival (OS), disease-free survival (DFS), and response to treatment.

## Patients and methods

### Ethics statement

The study was performed in accordance with the Good Clinical Practice and the Declaration of Helsinki and approved by the AVR Ethics Committee (protocol L3P2210, GifT). All the patients signed informed consent for the genetic analyses and the use of the results for research purposes.

### Patients and samples

We enrolled 144 consecutive withpatients TNBC (years 2019-2021) previously subjected to standard germline *BRCA1/2* testing and resulted wild type (WT) for the presence of alterations in the coding regions, after selection by the Genetic Counseling Service of IRCCS IRST.

The following data were collected from all consenting patients after registration:

- Demographic data: birthday, weight and height at the time of treatment initiation, ECOG performance status;
- Tumor information: date of diagnosis, cancer histology, grade and stage, date of second malignancy onset, type of tumor and its main characteristics;
- Treatment information: use of neoadjuvant treatment, date of start and end of chemotherapy, chemotherapeutic regimen with doses, number of cycles administered, type of surgery, date of surgery, date of progression/relapse (if any), number and types of further therapeutic regimens;
- Date of death or last follow-up (if still alive).

### Next-generation sequencing

All the experiments were performed in the IRST Biosciences Laboratory

Blood was stored at − 80°C until genomic DNA was extracted. DNA was purified by QIAamp DNA Mini Kit (Qiagen) and quantified using Qubit fluorometer (Thermo Fisher Scientific) with Qubit dsDNA BR Assay Kit.

Sequencing libraries were created starting from 200 ng of genomic DNA, following the protocol Illumina DNA Prep with Enrichment (Illumina) with an NGS custom panel (Integrated DNA Technologies).

The custom panel was designed including the promoter regions of 28 genes associated with a predisposition towards BOCs, retrieving the genomic coordinates of the promoters from UCSC and Ensembl genome browsers (**Table 1**). Sequencing was performed by using the NextSeq550 platform (Illumina) with NextSeq 500/550 Mid Output Kit v2.5 (300 Cycles) configured 2 × 151 cycles.

**Table 1.** NGS custom panel.

Gene	Chromosome	Transcript	Promoter coordinates (hg19)		
*ABRAXAS1*	4q21.23	NM_139076.3	84405004	–	84407579
*ATM*	11q22.3	NM_000051.4	108090352	–	108102824
*BAP1*	3p21.1	NM_004656.4	52443528	–	52445254
*BARD1*	2q35	NM_000465.4	215670586	–	215676228
*BLM*	15q26.1	NM_000057.4	91259223	–	91261915
*BRCA1*	17q21.31	NM_007294.4	41275950	–	41279128
*BRCA2*	13q13.1	NM_000059.4	32888279	–	32891522
*BRIP1*	17q23.2	NM_032043.3	59938392	–	59942231
*CDH1*	16q22.1	NM_004360.5	68770451	–	68781341
*CHEK2*	22q12.1	NM_007194.4	29136832	–	29139889
*MLH1*	3p22.2	NM_000249.4	37029343	–	37036702
*MRE11*	11q21	NM_005591.4	94225645	–	94228482
*MSH2*	2p21-p16.3	NM_000251.3	47628819	–	47632070
*MSH6*	2p16.3	NM_000179.3	48008831	–	48012262
*NBN*	8q21.3	NM_002485.5	90992947	–	90997773
*NF1*	17q11.2	NM_001042492.3	29420791	–	29423873
*NTHL1*	16p13.3	NM_002528.7	2096199	–	2099088
*PALB2*	16p12.2	NM_024675.4	23650914	–	23654441
*PMS2*	7p22.1	NM_000535.7	6046956	–	6053558
*PTEN*	10q23.31	NM_000314.8	89619851	–	89629136
*RAD50*	5q31.1	NM_005732.4	131891483	–	131895115
*RAD51C*	17q22	NM_058216.3	56768997	–	56771624
*RAD51D*	17q12	NM_002878.4	33445311	–	33447640
*RECQL4*	8q24.3	NM_004260.4	145741765	–	145744684
*STK11*	19p13.3	NM_000455.5	1202623	–	1210888
*TP53*	17p13.1	NM_000546.6	7587180	–	7593026
*WRN*	8p12	NM_000553.6	30890166	–	30893433
*XRCC2*	7q36.1	NM_005431.2	152371781	–	152374441

List of the 28 cancer predisposition genes included in the NGS custom panel with the chromosome position, the name of the transcript and the genomic coordinates of the promoter regions analyzed.

### Bioinformatics analysis

Raw reads were analyzed with Illumina DRAGEN Bio-IT Platform v4.0 (1), on the hg19 reference genome, using the following command line parameters:

-- read-trimmers quality,adapter --trim-min-quality 20 --trim-adapter-read1 < adapter_file> --trim-adapter-read2 < adapter_file>
-- enable-duplicate-marking true
-- enable-variant-caller true
-- vc-target-bed < bed_file>
-- vc-target-bed-padding 50

Resulting variant calls were annotated with ANNOVAR v2020-06-07 (2) on the databases refGene, gnomad211_exome, clinvar_20221231. Then, the variants in the promoters were manually filtered with the following exclusion criteria:

- Variants present in >5 patients;
- Variants with a population frequency >5% (based on the gnomAD database);
- Variants with a variant allele frequency (VAF) <0.3 (considered unreliable calls on germline DNA);
- Variants with a coverage <10 (deemed unreliable calls);
- Variants classified as benign/likely benign on ClinVar;
- Variants located in highly repetitive regions (microsatellites).

The remaining variants were used to produce Oncoprint plots (R package ComplexHeatmap v2.16.0) and Lolliplots (R package trackViewer v1.38.1).

### Statistical analysis

Median values (with interquartile range-IQR) were reported for continuous variables, while absolute values and percentages were reported for non-continuous variables. DFS was calculated in months as the difference between the date of diagnosis and the date of disease progression for patients experiencing progression. For patients who did not experience progression or had no evidence of disease at the time of death, DFS was calculated as the difference between the date of diagnosis and the date of the last disease re-evaluation or the date of death. Events were defined as instances of disease progression or deaths without evidence of disease. OS was calculated in months as the difference between the date of diagnosis and the date of death for deceased patients. For living patients, OS was calculated as the difference between the date of diagnosis and the date of the last follow-up. Events were represented by deaths. The association between clinical characteristics and genetic variants was assessed using the Chi-square test. The probability percentages for DFS and OS were calculated using the Kaplan-Meier product limit method.^13^ The proportional hazards Cox regression model was used to calculate Hazard Ratios (HR) and their corresponding 95% confidence intervals (95% CI) for covariates, both in univariate and multivariate analyses. Due to the exploratory nature of the study, no multiple testing corrections were made. All *P*-values were determined using two-tailed tests, and the statistical analyses were conducted using the SAS statistical software, version 9.4 (SAS Institute).

## Results

Between January 2019 and December 2021, 144 patients with TNBC were recruited in this study.

Complete clinical characteristics were available for 75 patients and are summarized in **Table 2**. Among the 75 patients, with a median age of 53 years (range, IQR 47-60), 29 (38.7%) had a family history of cancer, 3 (4%) presented bilateral tumors, 22 (29.3%) had residual disease after neoadjuvant chemotherapy, only 3 patients (4%) developed a second tumor. Most of the patients received anthracycline therapy in a (neo) adjuvant setting (85.3%); relapse occurred in 22 patients (29%).

**Table 2.** Patient clinical characteristics.

	*N*. (%)
Age: median value (range, IQR)	53 (34-71, 47-60)
Family history of cancer	
No	46 (61.3)
Yes	29 (38.7)
Bilateral tumors	
No	72 (96.0)
Yes	3 (4.0)
Stage	
I	25 (33.3)
II	31 (41.4)
III	19 (25.3)
Anthracycline therapy:	
No	11 (14.7)
Yes	64 (85.3)
Neoadjuvant chemotherapy	
No	42 (56.0)
Yes	33 (44.0)
Pathological complete response (pCR)	
Yes	11 (33.0)
No	22 (67.0)
Adjuvant chemotherapy	
No	23 (30.7)
Yes	52 (69.3)

List of the patient’s clinical characteristics. The table shows family history of cancer, presence of tumor bilaterality, tumor stage, treatment data, and response to therapy. N: number, IQR: interquartile range.

**Figure 2.**
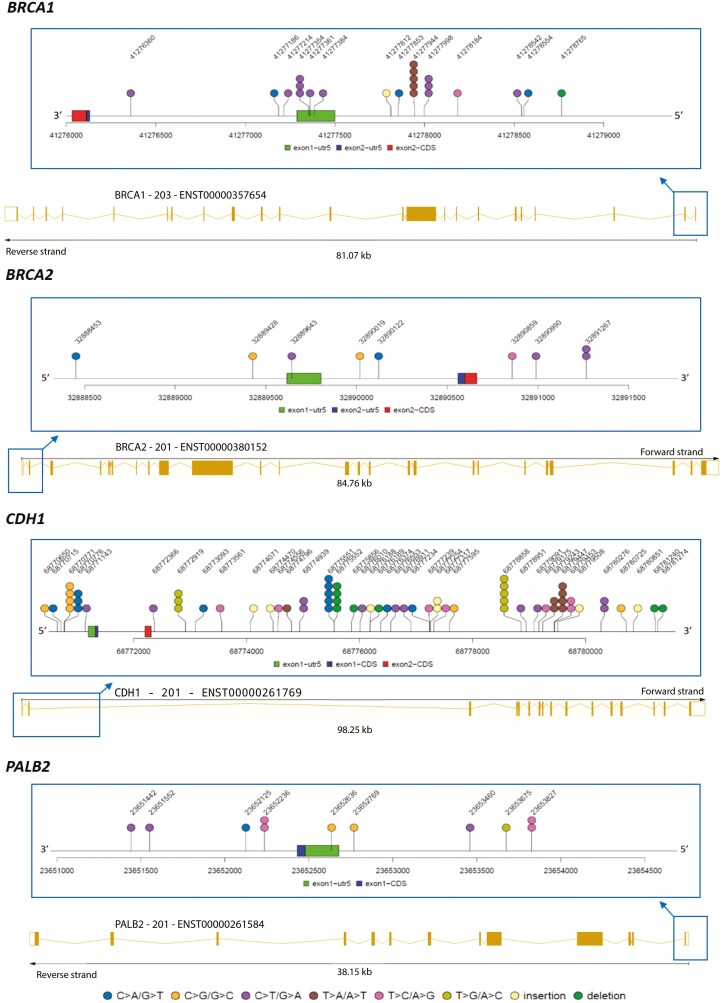
Lolliplots showing the position of alterations identified in regulatory regions of *BRCA1*, *BRCA2*, *CDH1* and *PALB2.* The plot range is the promoter region of each gene (see Table 1), with a downstream padding of 300 bp.

The NGS analysis on the promoter regions of 28 genes of the 144 patients with TNBC, revealed the presence of 635 rare variants: 54 small deletions, 28 small insertions and 553 nucleotide changes, as shown in **Supplementary Table S1**. Out of these 635 rare variants, the most frequently alterations were in the promoters of the *CDH1* (40%), *ATM* (35%), *STK11* (31%), *PTEN* (18%), *PMS2* (21%), *MSH2* (15%), and *BARD1* (16%) genes (**Supplementary Figure S1**). A more in-depth analysis in regulatory regions of 4 genes of interest was conducted to highlight the position of alterations (**Figure 2**).

**Figure 3.**
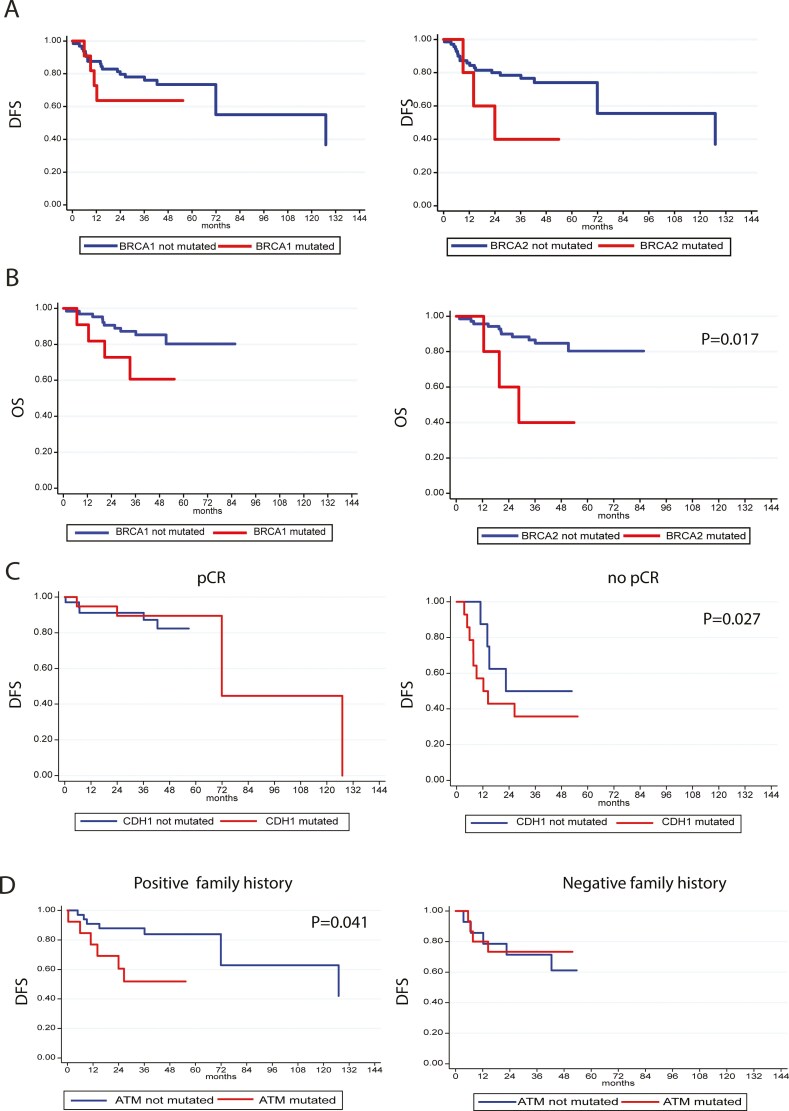
(A) Disease-free survival (DFS) according to *BRCA1/2* non-coding regions status: on the left DFS and *BRCA1*non-coding regions: on the right DFS and regulatory regions of *BRCA2*. (B) Overall survival (OS) according to *BRCA1/2* non-coding regions status on the left relationship between alterated non-coding regions of *BRCA1* and OS, on the right relationship between regulatory regions of *BRCA2* and OS (C) DFS according to non-coding regions of *CDH1* status and pathological complete response (pCR): on the left pCR, on the right no pCR. (D) DFS according to *ATM* status in regulatory regions and family history: on the left Positive family history; on the right Negative family history.

Rare germline variants in *BRCA2* promoter were statistically significantly related to worse DFS (**Figure 3A**) and OS (HR = 4.76, 95% CI, 1.32-17.15, *P* = .017) (**Figure 3B**). No differences in DFS and OS were found for other genes. Rare variants in *CDH1* promoter were related to the highest percentage of non-pathological complete response (pCR) (*P* = .027) (**Figure 3C**). *R*are variants in *MLH1* and *PALB2* promoters were found to be related to bilateral BC (*P* = .015 and *P* < 0.001, respectively—data not shown). Rare variants of the regulatory regions in *ATM* gene were associated with a positive family history (*P* = .041), as shown in **Figure 3D**.

As regards the patient clinical characteristics, the stage at diagnosis and the residual disease were unfavorable prognostic factor for both univariate DFS and OS (**Table 3**).

**Table 3.** Univariate analysis of DFS and OS according to clinical characteristics

	DFS		OS	
	HR (95% CI)	*P*	HR (95% CI)	*P*
Family history of cancer				
No	1.00		1.00	
Yes	1.34 (0.56-3.24)	.511	0.88 (0.29-2.63)	.822
Bilateral tumors				
No	1.00		1.00	
Yes	NE	–	NE	–
Residual disease				
No	1.00		1.00	
Yes	6.09 (2.41-15.38)	.0001	3.80 (1.31-10.98)	.014
Stage				
I	1.00		1.00	
II	3.57 (0.99-12.90)		NE	
III	7.42 (1.94-28.42)	.013	NE	–

Univariate analysis of DFS and OS according to clinical characteristics.

Abbreviations: DFS, disease -free survival; NE, not estimable, OS, overall survival.

In multivariate analysis, including *BRCA2* promoter, residual disease, and age, after backward stepwise selection, rare germline variants of *BRCA2* promoter are confirmed to be an independent prognostic factor correlated with shorter DFS (HR = 4.39, 95% CI, 1.17-16.52, *P* = .028) and OS (HR = 6.23, 95% CI, 1.49-26.09, *P* = 0.012) (**Table 4**).

**Table 4.** Multivariate analysis of DFS and OS according to age, residual disease, and germline *BRCA2* rare variants.

	DFS		OS	
	HR (95% CI)	*P*	HR (95% CI)	*P*
Residual disease				
No	1.00		1.00	
Yes	4.41 (1.64-11.89)	.003	3.46 (1.03-11.65)	.045
*BRCA2*				
WT	1.00		1.00	
mutated	4.39 (1.17-16.52)	.028	6.23 (1.49-26.09)	.012

Abbreviations: DFS, disease-free survival; OS, overall survival; WT, wild type.

## Discussion

Inherited pathogenic variants in the *BRCA1* and *BRCA2* genes are well-established risk factors for BC, particularly in women with a family history of the disease.^14^ TNBC is an aggressive subtype of BC that is associated with a poorer prognosis compared to other subtypes.^13^ Recent studies have shown that rare germline variants in non-coding regions of *BRCA1/2* and other genes can contribute to the development of the disease.^8,9,14^ Traditional genetic screening methods, such as those used in commercial tests (eg, FoundationOne), focus primarily on exonic regions, which may miss significant non-coding variants that can affect gene expression and contribute to cancer risk.^9^ Emerging evidence suggests that variants in these regulatory regions may influence not only the likelihood of developing BC, but also treatment response and overall clinical outcomes.^15,16^ This highlights the need to expand genetic testing to include regulatory regions, as understanding these elements could lead to more comprehensive risk assessments and personalized treatment strategies, ultimately improving patient management and outcomes. In particular, a recent study has shown that variants in the 5′ region of *BRCA1* and *BRCA2* genes can alter promoter activity and protein binding, which can affect gene expression, ultimately impacting BC risk.^8^ Another study found that non-coding variants in *BRCA1* and *BRCA2* genes can affect the splicing of RNA, which can lead to the production of abnormal proteins and contribute to cancer development.^17^ These variants are spread throughout the regulatory and promoter regions of the genes and it can be difficult to detect them using traditional genetic testing methods.^18^ Moreover, there is limited information on ongoing clinical trials investigating the impact of non-coding variants in *BRCA1* and *BRCA2* regulatory regions on BC prognosis. While these studies suggest that variants in *BRCA1* and *BRCA2* genes can impact BC risk, there is limited research on their impact on BC prognosis.^19,20^ To date, the risks associated with rare variants in BC predisposition genes have been largely unclear.

We aimed to investigate the impact of rare germline variants in promoter regions of *BRCA1/2* and other 26 cancer predisposition genes on TNBC. In our series of 144 patients with early TNBC previously tested WT for the presence of germline variants in the coding regions of *BRCA1/2* and other cancer predisposition genes, all patients were found to have at least one rare variant in the promoter regions of 28 BC predisposition genes. In line with the literature data, among the most altered regulatory regions we identified mainly genes involved in tumor suppression and DNA damage repair systems.^21,22^ For 75 patients, complete clinical data were available and we correlated the presence of the rare variants in the regulatory regions of 28 genes with the clinical variables of interest and disease aggressiveness. Rare non-coding germline variants in *BRCA2* promoter were found to significantly worsen OS (*P* = .017; HR = 4.76, 95% CI, 1.32-17.15). In the POSH study,^23^ patients with a *BRCAmut* had a similar prognosis as patients without these alterations, but several studies have indicated that variants in *BRCA1* and *BRCA2* may impact the effectiveness of chemotherapy. The GeparSixto Study^24^ randomly assigned patients to either standard chemotherapy containing anthracycline and taxane alone or with the addition of carboplatin. The trial demonstrated that in patients with TNBC, the presence of a *BRCA1/2* variant was linked to a higher percentage of patients achieving pCR and improved survival when compared to those without alterations, irrespective of other factors (eg, chemotherapy). Patients without *BRCA1/2* alterations who received standard chemotherapy without carboplatin had lower DFS rates than those who received chemotherapy plus carboplatin.^25^ Therefore, patients with a *BRCAmut* TNBC might have a survival advantage because of the higher efficacy of systemic chemotherapy. The GeparOLA study^26^ aimed to assess the efficacy and safety of neoadjuvant olaparib in combination with paclitaxel compared to carboplatin in patients with HER2-negative BC and homologous recombination deficiency. Although the study did not meet its primary endpoint, it demonstrated that olaparib in a neoadjuvant setting is comparable to carboplatin for patients with a *BRCAmut* while exhibiting reduced toxicity. This finding supports the notion that by continuing to select patients who are sensitive to PARPi, there is potential for a de-escalation strategy regarding treatment toxicity for a broader patient population. None of our patients received platinum salts as chemotherapy. In addition, an Italian study observed a lower BC-specific OS rate in *BRCA2* variant carriers after the first two years after diagnosis.^27^ Most of the deaths in our case series were observed in the first 2 years from diagnosis. Consistent with literature data, rare variants in regulatory region of *PALB2* were present in 10% of patients, and 29% of the carriers had bilateral tumors with a positive statistical association (*P* < .001).^28^ Prophylactic bilateral mastectomy is indicated in several clinical scenarios for women with *PALB2* mutations, particularly when BC is identified in one breast. This surgical procedure has the potential to lower the risk of developing neoplasms in the contralateral breast by as much as 95%. The National Comprehensive Cancer Network guidelines advocate that individuals with *PALB2* mutations should undergo genetic counseling to assess the advantages of bilateral mastectomy along with other risk-reducing strategies. These recommendations emphasize the importance of a personalized approach to cancer risk management, considering both individual and familial cancer histories.^28^

Similarly, *MLH1* regulatory variants were found in 16% of our cases and were related to a higher risk of bilateral tumors (*P* = .015). *MLH1* gene, involved in Lynch syndrome plays a significant role in cancer predisposition, since *MLH1* mutation carriers have a high risk for multiple primary cancers, including colorectal, endometrial, ovarian and breast cancers.^29,30^ Moreover, variants in *ATM* regulatory regions were strongly associated with a positive family history (*P* = 0.041) whilst alterations in the regulatory region of *CDH1* were strongly associated with residual after neoadjuvant chemotherapy (*P* = .027) being present in 44% of all cases. In the EpiTax-trial, *CDH1* mutations predicted an inferior response in the paclitaxel arm (*P* = .01) as well as the epirubicin arm (*P* = .04).^31^ Our findings, together with literature data, support the predictive value of *CDH1* mutations in relation to treatment outcomes after neoadjuvant chemotherapy, which aligns the significance of alterations also in the regulatory non-coding region of *CDH1* and residual disease.

Overall, our results underline the importance of extended genetic testing using panels including the promoter regions of genes involved in cancer predisposition, especially in BC patients lacking variants in the coding regions of *BRCA1/2.* The last American College of Medical Genetics guidelines^32^ do not provide specific recommendations for the reporting and classification of variants identified in *BRCA1/2* promoter, intronic, and untranslated regions. Therefore, carriers should be managed exclusively based on their personal and family history, which allows for the estimation of cancer risk. The identification of genetic variants in the regulatory regions of cancer predisposition genes could modify the clinical, personal, and familial history in terms of surveillance, prevention strategies, and also personalized treatments, such as the use of PARP inhibitors in patients with alterations in homologous recombination genes. Variants of uncertain significance represent a challenge, since the disease risk associated with them may be over-interpreted or misinterpreted. As a result, it should not be used for clinical decision-making. To date, there is limited available data concerning sequence alterations in non-coding regions of *BRCA1/2*. Even less information is available about the outcome of carriers who should be managed based on their lifetime cancer risk once their genetic screening remains inconclusive. In the future, the use of genetic tests including the regulatory and non-coding regions of cancer predisposition genes will improve the management and treatment of patients with cancer predisposition.

## Conclusion

To summarize, our study underscores the growing significance of rare germline variants in the regulatory regions of genes such as *BRCA1/2* in contributing to TNBC predisposition. Identifying individuals at increased risk due to these variants can guide clinical management, potentially improving patient outcomes. Our study highlighted the importance of analyzing the non-coding and regulatory regions of cancer predisposition genes in patients with a suspected hereditary tumor. The associations identified between genetic alterations and clinical characteristics resulted to be statistically weak, due to the limited number of patients. However, the trends observed were in line with the literature data on carriers of variants in the coding regions of cancer predisposition genes, suggesting that also variants in non-coding regions can affect the gene function. Further research is necessary to fully understand their role in BC risk and to develop enhanced screening and prevention approaches for at-risk individuals. Given the limitations, our analyses should be regarded as preliminary, and larger studies are needed to validate these findings, including functional assays on the biological role of the identified alterations.

## Supplementary Material

oyaf052_suppl_Supplementary_Tables_1

oyaf052_suppl_Supplementary_Figures_1

## Data Availability

The data that support the findings of this study are available in the supplementary material of this article, and additional data are available upon request. No restrictions apply.
