# Supplementary material for: Exploring the promoter regions of cancer predisposition genes in patients with triple-negative breast cancer reveals the presence of rare germline variants
Source: Oncologist. 2025 May 8;30(5):oyaf052. doi: 10.1093/oncolo/oyaf052 (PMC12060721; doi:10.1093/oncolo/oyaf052)
Supplement: oyaf052_suppl_Supplementary_Tables_1 [file oyaf052_suppl_supplementary_tables_1.docx]

**Supplementary Table 1: List of the 635 rare variants identified in the 144 TNBC patients**

| **Sample ID** | **Chr** | **Start** | **End** | **Reference** | **Alternative** | **Variant type** | **dbSNP** | **Promoter region** | **ACMG (classification)** | **gnomAD** | **ClinVar** |
| --- | --- | --- | --- | --- | --- | --- | --- | --- | --- | --- | --- |
| TNBC0964 | chr17 | 56769496 | 56769496 | - | C | insertion |  | *RAD51C* | Likely Benign |  |  |
| TNBC0964 | chr19 | 1208337 | 1208337 | C | T | transition | rs967977236 | *STK11* | Likely Benign |  |  |
| TNBC0968 | chr15 | 91259413 | 91259413 | T | G | transversion | rs189455942 | *BLM* | Likely Benign |  |  |
| TNBC0968 | chr15 | 91259922 | 91259922 | G | A | transition |  | *BLM* | Likely Benign | 3.2e-05 |  |
| TNBC0968 | chr8 | 145743701 | 145743701 | G | A | transition | rs35697811 | *RECQL4* | Likely Benign | 0.21 |  |
| TNBC0973 | chr16 | 68775551 | 68775551 | C | A | transversion | rs79846694 | *CDH1* | Likely Benign | 4.24 |  |
| TNBC0973 | chr17 | 7587847 | 7587847 | A | T | transversion | rs753643217 | *TP53* | Likely Benign | 3.11 |  |
| TNBC0973 | chr2 | 47631288 | 47631289 | TG | - | deletion | rs17217737 | *MSH2* | Benign | 3.05 |  |
| TNBC0973 | chr2 | 47631791 | 47631791 | T | C | transition | rs72884632 | *MSH2* | Benign | 3.01 |  |
| TNBC0987 | chr17 | 7591436 | 7591436 | T | C | transition |  | *TP53* | Likely Benign |  |  |
| TNBC0998 | chr11 | 108102325 | 108102325 | A | G | transition | rs753929693 | *ATM* | Likely Benign | 12.17 |  |
| TNBC0998 | chr13 | 32888453 | 32888453 | C | A | transversion |  | *BRCA2* | Likely Benign |  |  |
| TNBC0998 | chr13 | 32890859 | 32890859 | A | G | transition |  | *BRCA2* | Likely Benign | 3.18e-05 |  |
| TNBC0998 | chr17 | 59941532 | 59941536 | AAAGA | - | deletion | rs528178795 | *BRIP1* | Benign | 2.37 |  |
| TNBC0999 | chr11 | 94227423 | 94227423 | G | A | transition | rs13447580 | *MRE11A* | Benign | 2.24 |  |
| TNBC0999 | chr16 | 68770776 | 68770776 | G | T | transversion | rs192929184 | *CDH1* | Likely Benign | 3.05 |  |
| TNBC0999 | chr16 | 68776374 | 68776374 | G | T | transversion |  | *CDH1* | Likely Benign |  |  |
| TNBC0999 | chr19 | 1203678 | 1203678 | T | C | transition | rs75605040 | *STK11* | Benign | 3.38 |  |
| TNBC0999 | chr19 | 1208855 | 1208855 | C | T | transition | rs113467487 | *STK11* | Benign | 3.23 |  |
| TNBC0999 | chr7 | 152371795 | 152371795 | T | - | deletion | rs369577931 | *XRCC2* | Likely Benign |  |  |
| TNBC0999 | chr8 | 30890961 | 30890961 | C | A | transversion | rs11574155 | *WRN* | Benign | 16.28 | Uncertain Significance |
| TNBC0999 | chr8 | 145744092 | 145744092 | C | T | transition | rs149515668 | *RECQL4* | Benign | 3.01 |  |
| TNBC1016 | chr16 | 68772919 | 68772919 | T | G | transversion | rs144910517 | *CDH1* | Likely Benign | 3.04 |  |
| TNBC1016 | chr17 | 41277853 | 41277853 | G | T | transversion | rs73625094 | *BRCA1* | Likely Benign | 3.37 |  |
| TNBC1021 | chr11 | 108099360 | 108099360 | A | G | transition | rs78326890 | *ATM* | Likely Benign | 14.52 |  |
| TNBC1021 | chr16 | 23653460 | 23653460 | G | A | transition | rs703771 | *PALB2* | Likely Benign | 10.38 |  |
| TNBC1021 | chr16 | 68780851 | 68780851 | - | AACA | insertion | rs201828383 | *CDH1* | Likely Benign |  |  |
| TNBC1021 | chr17 | 41278765 | 41278786 | GAGCCCTTCAGCCCGCCACTGC | - | deletion | rs149765996 | *BRCA1* | Likely Benign | 8.27 |  |
| TNBC1021 | chr2 | 215673243 | 215673243 | C | G | transversion | rs1037092702 | *BARD1* | Benign | 3.11 |  |
| TNBC1021 | chr22 | 29137902 | 29137902 | A | G | transition | rs576963837 | *CHEK2* | Likely Benign |  |  |
| TNBC1021 | chr3 | 52444872 | 52444872 | C | T | transition | rs566829817 | *BAP1* | Likely Benign | 9.34 |  |
| TNBC1026 | chr16 | 23653675 | 23653675 | A | C | transversion |  | *PALB2* | Likely Benign |  |  |
| TNBC1026 | chr17 | 41277998 | 41277998 | G | A | transition | rs73625095 | *BRCA1* | Benign | 2.07 |  |
| TNBC1026 | chr3 | 37031840 | 37031840 | A | C | transversion |  | *MLH1* | Likely Benign | 3.19e-05 |  |
| TNBC1026 | chr7 | 6051283 | 6051283 | A | G | transition | rs78848166 | *PMS2* | Benign | 2.19 |  |
| TNBC1034 | chr11 | 108096103 | 108096103 | G | T | transversion | rs530134971 | *ATM* | Likely Benign | 5.18 |  |
| TNBC1034 | chr5 | 131893898 | 131893898 | T | C | transition | rs191149532 | *RAD50* | Likely Benign | 12.44 |  |
| TNBC1034 | chr7 | 6052727 | 6052727 | C | T | transition | rs146016964 | *PMS2* | Likely Benign | 14.53 |  |
| TNBC1034 | chr7 | 152371795 | 152371795 | T | - | deletion | rs369577931 | *XRCC2* | Likely Benign |  |  |
| TNBC1034 | chr8 | 145743205 | 145743205 | G | T | transversion | rs35667555 | *RECQL4* | Benign | 3.31 |  |
| TNBC1035 | chr10 | 89627667 | 89627667 | A | T | transversion | rs78815234 | *PTEN* | Benign | 0.11 |  |
| TNBC1035 | chr11 | 108099374 | 108099374 | G | A | transition | rs79866170 | *ATM* | Benign | 2.22 |  |
| TNBC1035 | chr11 | 108101202 | 108101202 | A | G | transition | rs527682873 | *ATM* | Likely Benign | 12.13 |  |
| TNBC1035 | chr19 | 1202837 | 1202837 | T | C | transition | rs565798295 | *STK11* | Likely Benign | 1.59 |  |
| TNBC1037 | chr16 | 68776553 | 68776553 | G | A | transition |  | *CDH1* | Likely Benign |  |  |
| TNBC1037 | chr17 | 7590762 | 7590762 | A | G | transition |  | *TP53* | Likely Benign |  |  |
| TNBC1037 | chr7 | 152371795 | 152371795 | T | - | deletion | rs369577931 | *XRCC2* | Likely Benign |  |  |
| TNBC1038 | chr17 | 41277998 | 41277998 | G | A | transition | rs73625095 | *BRCA1* | Benign | 2.07 |  |
| TNBC1038 | chr3 | 37031336 | 37031336 | A | G | transition | rs141275247 | *MLH1* | Likely Benign | 6.38 |  |
| TNBC1038 | chr3 | 37035805 | 37035805 | A | G | transition | rs73826932 | *MLH1* | Benign | 3.09 |  |
| TNBC1038 | chr3 | 37036297 | 37036297 | - | T | insertion | rs113094640 | *MLH1* | Benign | 14.34 |  |
| TNBC1038 | chr5 | 131893261 | 131893261 | G | A | transition |  | *RAD50* | Likely Benign |  |  |
| TNBC1038 | chr8 | 145744092 | 145744092 | C | T | transition | rs149515668 | *RECQL4* | Benign | 3.01 |  |
| TNBC1051 | chr11 | 108096285 | 108096285 | C | T | transition | rs4987889 | *ATM* | Benign | 3.09 |  |
| TNBC1051 | chr16 | 23653827 | 23653827 | A | G | transition | rs548162840 | *PALB2* | Likely Benign | 2.52 |  |
| TNBC1051 | chr16 | 68775551 | 68775551 | C | A | transversion | rs79846694 | *CDH1* | Likely Benign | 4.24 |  |
| TNBC1051 | chr17 | 41278184 | 41278184 | T | C | transition | rs922274793 | *BRCA1* | Likely Benign |  |  |
| TNBC1051 | chr17 | 59941018 | 59941018 | A | - | deletion | rs559771069 | *BRIP1* | Likely Benign | 11.06 |  |
| TNBC1051 | chr2 | 215674657 | 215674657 | A | G | transition | rs753813308 | *BARD1* | Likely Benign | 10.14 |  |
| TNBC1054 | chr10 | 89619853 | 89619853 | A | C | transversion | rs183638448 | *PTEN* | Likely Benign | 4.14 |  |
| TNBC1054 | chr11 | 108102325 | 108102325 | A | G | transition | rs753929693 | *ATM* | Likely Benign | 12.17 |  |
| TNBC1054 | chr16 | 68775551 | 68775551 | C | A | transversion | rs79846694 | *CDH1* | Likely Benign | 4.24 |  |
| TNBC1054 | chr3 | 37031931 | 37031931 | A | G | transition | rs770219447 | *MLH1* | Benign | 2.27 |  |
| TNBC1065 | chr19 | 1210121 | 1210121 | A | T | transversion | rs148343995 | *STK11* | Benign | 3.47 |  |
| TNBC1065 | chr5 | 131893782 | 131893782 | G | A | transition | rs78524273 | *RAD50* | Benign | 3.17 |  |
| TNBC1079 | chr11 | 108092303 | 108092303 | C | G | transversion | rs4987874 | *ATM* | Benign | 2.16 |  |
| TNBC1079 | chr19 | 1205196 | 1205196 | C | - | deletion | rs538652196 | *STK11* | Likely Benign | 15.18 |  |
| TNBC1079 | chr19 | 1205197 | 1205197 | C | T | transition | rs559847307 | *STK11* | Likely Benign | 15.17 |  |
| TNBC1079 | chr7 | 6047135 | 6047135 | C | G | transversion | rs191289375 | *PMS2* | Likely Benign | 0.35 |  |
| TNBC1079 | chr8 | 30890961 | 30890961 | C | A | transversion | rs11574155 | *WRN* | Benign | 16.28 | Uncertain Significance |
| TNBC1079 | chr8 | 90994199 | 90994199 | T | C | transition | rs104895038 | *NBN* | Likely Benign | 1.51 | Not Provided |
| TNBC1089 | chr13 | 32890019 | 32890019 | C | G | transversion |  | *BRCA2* | Likely Benign |  |  |
| TNBC1089 | chr16 | 68775551 | 68775551 | C | A | transversion | rs79846694 | *CDH1* | Likely Benign | 4.24 |  |
| TNBC1089 | chr19 | 1205202 | 1205202 | T | C | transition | rs147492468 | *STK11* | Benign | 2.21 |  |
| TNBC1089 | chr2 | 48011758 | 48011758 | C | T | transition | rs766261325 | *MSH6* | Likely Benign |  |  |
| TNBC1089 | chr3 | 37030572 | 37030572 | T | A | transversion |  | *MLH1* | Likely Benign |  |  |
| TNBC1099 | chr16 | 68770771 | 68770771 | C | G | transversion | rs117397448 | *CDH1* | Likely Benign | 10.28 |  |
| TNBC1099 | chr17 | 7591514 | 7591514 | - | A | insertion | rs199831002 | *TP53* | Likely Benign |  |  |
| TNBC1099 | chr2 | 215671394 | 215671394 | T | C | transition |  | *BARD1* | Likely Benign | 3.19e-05 |  |
| TNBC1111 | chr10 | 89620174 | 89620174 | G | A | transition | rs80005718 | *PTEN* | Likely Benign | 4.53 |  |
| TNBC1111 | chr10 | 89620586 | 89620586 | G | C | transversion | rs183894316 | *PTEN* | Likely Benign | 9.01 |  |
| TNBC1111 | chr10 | 89622647 | 89622647 | G | A | transition | rs115389083 | *PTEN* | Benign | 0.14 |  |
| TNBC1111 | chr11 | 94227087 | 94227087 | G | A | transition | rs13447582 | *MRE11A* | Benign | 10.34 |  |
| TNBC1111 | chr11 | 108096863 | 108096863 | T | - | deletion | rs4987892 | *ATM* | Benign | 1.59 |  |
| TNBC1111 | chr11 | 108102150 | 108102150 | C | T | transition | rs115579447 | *ATM* | Benign | 1.58 |  |
| TNBC1111 | chr15 | 91259609 | 91259609 | G | C | transversion | rs7178640 | *BLM* | Benign | 5.48 |  |
| TNBC1111 | chr15 | 91259989 | 91259992 | AAGT | - | deletion | rs142264462 | *BLM* | Benign | 3.56 |  |
| TNBC1111 | chr15 | 91261640 | 91261640 | G | A | transition | rs76758344 | *BLM* | Benign | 3.36 |  |
| TNBC1111 | chr16 | 68775856 | 68775857 | GA | - | deletion | rs35993652 | *CDH1* | Likely Benign | 7.29 |  |
| TNBC1111 | chr16 | 68867074 | 68867074 | C | T | transition | rs116206419 | *CDH1* | Benign | 1.12 |  |
| TNBC1111 | chr17 | 29423156 | 29423156 | C | A | transversion | rs146629985 | *NF1* | Benign | 15.03 |  |
| TNBC1111 | chr17 | 41277812 | 41277812 | - | G | insertion | rs144689288 | *BRCA1* | Likely Benign |  |  |
| TNBC1111 | chr2 | 47629718 | 47629718 | G | T | transversion | rs17224080 | *MSH2* | Likely Benign | 4.44 |  |
| TNBC1111 | chr7 | 6053465 | 6053465 | C | T | transition | rs189092086 | *PMS2* | Likely Benign | 3.05 |  |
| TNBC1111 | chr7 | 152373346 | 152373346 | A | G | transition | rs542356939 | *XRCC2* | Likely Benign | 14.53 |  |
| TNBC1121 | chr16 | 23652125 | 23652125 | G | T | transversion | rs142098064 | *PALB2* | Benign | 7.48 |  |
| TNBC1121 | chr2 | 48011645 | 48011645 | A | G | transition | rs114144926 | *MSH6* | Benign | 2.43 |  |
| TNBC1121 | chr3 | 37036555 | 37036555 | G | A | transition | rs567565350 | *MLH1* | Likely Benign | 3.43 |  |
| TNBC1121 | chr7 | 6049428 | 6049429 | AG | - | deletion | rs940383307 | *PMS2* | Likely Benign | 7.21 |  |
| TNBC1121 | chr8 | 90995348 | 90995350 | AAA | - | deletion |  | *NBN* | Uncertain Significance |  |  |
| TNBC1129 | chr17 | 7587474 | 7587474 | T | C | transition | rs187658938 | *TP53* | Likely Benign | 8.27 |  |
| TNBC1129 | chr17 | 29421869 | 29421869 | A | C | transversion | rs17879128 | *NF1* | Benign | 2.28 |  |
| TNBC1129 | chr5 | 131893782 | 131893782 | G | A | transition | rs78524273 | *RAD50* | Benign | 3.17 |  |
| TNBC1143 | chr16 | 68774071 | 68774071 | - | T | insertion | rs749175536 | *CDH1* | Likely Benign |  |  |
| TNBC1143 | chr2 | 47631089 | 47631089 | G | C | transversion |  | *MSH2* | Likely Benign |  |  |
| TNBC1146 | chr10 | 89626845 | 89626845 | A | T | transversion | rs41284070 | *PTEN* | Benign | 3.18 |  |
| TNBC1146 | chr16 | 23653827 | 23653827 | A | G | transition | rs548162840 | *PALB2* | Likely Benign | 2.52 |  |
| TNBC1146 | chr8 | 145743269 | 145743269 | G | A | transition | rs36089568 | *RECQL4* | Likely Benign | 5.43 |  |
| TNBC1146 | chr8 | 145743874 | 145743874 | C | T | transition | rs34473676 | *RECQL4* | Likely Benign | 5.38 |  |
| TNBC1155 | chr10 | 89623766 | 89623766 | G | A | transition |  | *PTEN* | Likely Benign | 0.14 | Conflicting Interpretations |
| TNBC1155 | chr17 | 29422027 | 29422027 | A | G | transition | rs1055865194 | *NF1* | Likely Benign |  |  |
| TNBC1155 | chr2 | 47631341 | 47631341 | C | G | transversion | rs773628858 | *MSH2* | Likely Benign |  |  |
| TNBC1155 | chr3 | 37030924 | 37030924 | G | A | transition | rs1020061560 | *MLH1* | Likely Benign |  |  |
| TNBC1161 | chr16 | 68778858 | 68778858 | A | C | transversion | rs150045763 | *CDH1* | Likely Benign | 7.13 |  |
| TNBC1161 | chr22 | 29137216 | 29137216 | T | A | transversion | rs146028137 | *CHEK2* | Likely Benign | 4.42 |  |
| TNBC1161 | chr5 | 131894351 | 131894351 | C | T | transition | rs891158702 | *RAD50* | Likely Benign |  |  |
| TNBC1169 | chr19 | 1207920 | 1207920 | C | T | transition | rs987791840 | *STK11* | Likely Benign |  |  |
| TNBC1169 | chr8 | 145743269 | 145743269 | G | A | transition | rs36089568 | *RECQL4* | Likely Benign | 5.43 |  |
| TNBC1169 | chr8 | 145743874 | 145743874 | C | T | transition | rs34473676 | *RECQL4* | Likely Benign | 5.38 |  |
| TNBC1169 | chr8 | 145744551 | 145744551 | C | T | transition |  | *RECQL4* | Likely Benign |  |  |
| TNBC1172 | chr13 | 32890990 | 32890990 | G | A | transition | rs11571575 | *BRCA2* | Likely Benign | 2.27 |  |
| TNBC1172 | chr16 | 68770776 | 68770776 | G | T | transversion | rs192929184 | *CDH1* | Likely Benign | 3.05 |  |
| TNBC1172 | chr17 | 41277998 | 41277998 | G | A | transition | rs73625095 | *BRCA1* | Benign | 2.07 |  |
| TNBC1172 | chr2 | 215671029 | 215671029 | C | T | transition | rs113594759 | *BARD1* | Benign | 2.41 |  |
| TNBC1177 | chr11 | 94227791 | 94227791 | A | C | transversion | rs555505826 | *MRE11A* | Likely Benign | 15.56 |  |
| TNBC1177 | chr11 | 108096618 | 108096618 | T | C | transition |  | *ATM* | Likely Benign |  |  |
| TNBC1177 | chr19 | 1203678 | 1203678 | T | C | transition | rs75605040 | *STK11* | Benign | 3.38 |  |
| TNBC1177 | chr19 | 1204770 | 1204770 | T | G | transversion | rs190616081 | *STK11* | Likely Benign | 10.17 |  |
| TNBC1177 | chr19 | 1205196 | 1205196 | C | - | deletion | rs538652196 | *STK11* | Likely Benign | 15.18 |  |
| TNBC1177 | chr19 | 1205197 | 1205197 | C | T | transition | rs559847307 | *STK11* | Likely Benign | 15.17 |  |
| TNBC1177 | chr19 | 1208855 | 1208855 | C | T | transition | rs113467487 | *STK11* | Benign | 3.23 |  |
| TNBC1177 | chr22 | 29137163 | 29137163 | T | C | transition | rs17885379 | *CHEK2* | Benign | 2.35 |  |
| TNBC1191 | chr17 | 7587687 | 7587687 | A | G | transition | rs76923748 | *TP53* | Benign | 1.46 |  |
| TNBC1191 | chr17 | 41277354 | 41277354 | G | A | transition | rs143160357 | *BRCA1* | Likely Benign | 2.39 | Conflicting Interpretations |
| TNBC1191 | chr7 | 152372673 | 152372673 | G | T | transversion | rs370903842 | *XRCC2* | Likely Benign |  |  |
| TNBC1206 | chr10 | 89619853 | 89619853 | A | C | transversion | rs183638448 | *PTEN* | Likely Benign | 4.14 |  |
| TNBC1206 | chr17 | 29421869 | 29421869 | A | C | transversion | rs17879128 | *NF1* | Benign | 2.28 |  |
| TNBC1206 | chr3 | 52444555 | 52444555 | A | - | deletion | rs1019466039 | *BAP1* | Likely Benign | 3.27e-05 |  |
| TNBC1217 | chr11 | 108091405 | 108091405 | G | T | transversion | rs185663398 | *ATM* | Likely Benign | 0.35 |  |
| TNBC1217 | chr16 | 68779447 | 68779447 | A | T | transversion |  | *CDH1* | Benign | 2.46 |  |
| TNBC1217 | chr17 | 41277354 | 41277354 | G | A | transition | rs143160357 | *BRCA1* | Likely Benign | 2.39 | Conflicting Interpretations |
| TNBC1217 | chr17 | 56769568 | 56769568 | T | G | transversion | rs568361156 | *RAD51C* | Likely Benign | 12.49 |  |
| TNBC1217 | chr19 | 1204816 | 1204816 | C | T | transition | rs144386622 | *STK11* | Benign | 5.43 |  |
| TNBC1217 | chr19 | 1204916 | 1204916 | A | G | transition | rs3795060 | *STK11* | Benign | 2.21 |  |
| TNBC1217 | chr19 | 1210654 | 1210654 | A | G | transition | rs57782778 | *STK11* | Benign | 6.11 |  |
| TNBC1217 | chr2 | 47629546 | 47629546 | A | C | transversion |  | *MSH2* | Likely Benign |  |  |
| TNBC1219 | chr10 | 89626056 | 89626056 | A | T | transversion | rs35930911 | *PTEN* | Likely Benign | 4.08 |  |
| TNBC1219 | chr11 | 108090462 | 108090462 | - | AAC | insertion |  | *ATM* | Likely Benign |  |  |
| TNBC1219 | chr11 | 108091177 | 108091177 | C | A | transversion | rs1442729 | *ATM* | Benign | 3.55 |  |
| TNBC1219 | chr11 | 108094738 | 108094738 | G | A | transition | rs147161304 | *ATM* | Likely Benign | 0.51 |  |
| TNBC1219 | chr11 | 108101838 | 108101838 | G | C | transversion | rs74706571 | *ATM* | Benign | 3.54 |  |
| TNBC1219 | chr16 | 68776188 | 68776188 | - | T | insertion |  | *CDH1* | Likely Benign |  |  |
| TNBC1219 | chr17 | 29422792 | 29422792 | C | T | transition | rs17878346 | *NF1* | Benign | 1.26 |  |
| TNBC1219 | chr17 | 56769213 | 56769213 | C | A | transversion |  | *RAD51C* | Likely Benign |  |  |
| TNBC1219 | chr17 | 56770740 | 56770740 | T | A | transversion |  | *RAD51C* | Likely Benign | 3.18e-05 |  |
| TNBC1219 | chr2 | 215670679 | 215670679 | A | G | transition | rs75136258 | *BARD1* | Benign | 16.08 |  |
| TNBC1219 | chr2 | 215671862 | 215671862 | C | T | transition | rs6752805 | *BARD1* | Benign | 2.37 |  |
| TNBC1219 | chr2 | 215673039 | 215673041 | AGG | - | deletion | rs377244276 | *BARD1* | Likely Benign | 3.21 |  |
| TNBC1219 | chr2 | 215674836 | 215674836 | C | T | transition | rs115285362 | *BARD1* | Likely Benign | 0.28 |  |
| TNBC1219 | chr2 | 215674905 | 215674905 | T | C | transition | rs73989372 | *BARD1* | Benign | 4.33 |  |
| TNBC1219 | chr3 | 37030229 | 37030229 | T | C | transition | rs4678557 | *MLH1* | Benign | 9.06 |  |
| TNBC1219 | chr3 | 37032356 | 37032356 | A | G | transition | rs62244262 | *MLH1* | Likely Benign | 7.26 |  |
| TNBC1219 | chr4 | 84405171 | 84405171 | C | T | transition | rs76009998 | *ABRAXAS1* | Benign | 3.58 |  |
| TNBC1219 | chr4 | 84405172 | 84405172 | C | T | transition | rs77408148 | *ABRAXAS1* | Benign | 4.51 |  |
| TNBC1219 | chr4 | 84405406 | 84405406 | T | C | transition | rs79273060 | *ABRAXAS1* | Benign | 3.59 |  |
| TNBC1219 | chr4 | 84406969 | 84406969 | G | C | transversion | rs80290794 | *ABRAXAS1* | Benign | 4.56 |  |
| TNBC1219 | chr7 | 6048070 | 6048070 | T | C | transition | rs753584548 | *PMS2* | Likely Benign | 6.38e-05 |  |
| TNBC1219 | chr7 | 152371846 | 152371847 | TA | - | deletion |  | *XRCC2* | Likely Benign | 4.79e-05 |  |
| TNBC1224 | chr11 | 108091405 | 108091405 | G | T | transversion | rs185663398 | *ATM* | Likely Benign | 0.35 |  |
| TNBC1224 | chr7 | 6052220 | 6052220 | A | T | transversion | rs148315304 | *PMS2* | Benign | 13.38 |  |
| TNBC1245 | chr10 | 89627667 | 89627667 | A | T | transversion | rs78815234 | *PTEN* | Benign | 0.11 |  |
| TNBC1245 | chr16 | 68824805 | 68824805 | T | A | transversion | rs200158271 | *CDH1* | Likely Benign | 3.24 |  |
| TNBC1246 | chr11 | 108093472 | 108093472 | C | - | deletion | rs569288775 | *ATM* | Likely Benign |  |  |
| TNBC1246 | chr16 | 68779447 | 68779447 | A | T | transversion |  | *CDH1* | Benign | 2.46 |  |
| TNBC1246 | chr17 | 29422516 | 29422516 | G | T | transversion | rs532398315 | *NF1* | Benign | 11.11 |  |
| TNBC1246 | chr22 | 29137095 | 29137095 | G | A | transition | rs140000715 | *CHEK2* | Likely Benign | 15.25 |  |
| TNBC1246 | chr22 | 29139189 | 29139189 | G | A | transition | rs553987644 | *CHEK2* | Likely Benign | 2.39 |  |
| TNBC1246 | chr7 | 6051275 | 6051275 | A | G | transition |  | *PMS2* | Likely Benign |  |  |
| TNBC1255 | chr10 | 89623766 | 89623766 | G | A | transition |  | *PTEN* | Likely Benign | 0.14 | Conflicting Interpretations |
| TNBC1255 | chr16 | 68773093 | 68773093 | G | T | transversion | rs913952654 | *CDH1* | Likely Benign |  |  |
| TNBC1255 | chr22 | 29137095 | 29137095 | G | A | transition | rs140000715 | *CHEK2* | Likely Benign | 15.25 |  |
| TNBC1255 | chr7 | 152374029 | 152374029 | C | T | transition | rs558913492 | *XRCC2* | Likely Benign | 3.19e-05 |  |
| TNBC1269 | chr11 | 108096240 | 108096240 | G | A | transition | rs4987888 | *ATM* | Likely Benign | 4.15 |  |
| TNBC1269 | chr17 | 7588068 | 7588068 | C | T | transition | rs533477844 | *TP53* | Likely Benign | 4.17 |  |
| TNBC1269 | chr2 | 47629296 | 47629296 | G | A | transition | rs964090784 | *MSH2* | Likely Benign | 3.19e-05 |  |
| TNBC1269 | chr2 | 47631290 | 47631290 | T | - | deletion | rs201729802 | *MSH2* | Benign | 13.36 |  |
| TNBC1269 | chr3 | 37030996 | 37030996 | T | C | transition | rs144367648 | *MLH1* | Likely Benign | 3.49 |  |
| TNBC1270 | chr17 | 33445732 | 33445732 | C | T | transition | rs567683181 | *RAD51D* | Benign | 10.05 |  |
| TNBC1270 | chr2 | 215676058 | 215676058 | C | T | transition | rs773718131 | *BARD1* | Likely Benign | 2.39 |  |
| TNBC1270 | chr7 | 6048012 | 6048012 | T | C | transition | rs554268167 | *PMS2* | Likely Benign | 3.19e-05 |  |
| TNBC1278 | chr19 | 1203095 | 1203095 | G | A | transition | rs993226336 | *STK11* | Likely Benign | 3.32e-05 |  |
| TNBC1278 | chr2 | 48011727 | 48011727 | G | T | transversion | rs114513560 | *MSH6* | Likely Benign | 10.08 |  |
| TNBC1278 | chr7 | 152373424 | 152373424 | G | T | transversion | rs1003140794 | *XRCC2* | Likely Benign |  |  |
| TNBC1282 | chr16 | 2096754 | 2096754 | C | T | transition |  | *NTHL1* | Likely Benign |  |  |
| TNBC1282 | chr16 | 23652236 | 23652236 | T | C | transition | rs141315496 | *PALB2* | Likely Benign | 3.43 |  |
| TNBC1282 | chr2 | 48011971 | 48011971 | A | T | transversion |  | *MSH6* | Likely Benign |  |  |
| TNBC1282 | chr7 | 6049597 | 6049597 | A | T | transversion |  | *PMS2* | Benign | 0.16 |  |
| TNBC1282 | chr7 | 6049620 | 6049620 | - | TATG | insertion |  | *PMS2* | Likely Benign |  |  |
| TNBC1287 | chr11 | 108095034 | 108095034 | G | A | transition | rs117590923 | *ATM* | Benign | 2.25 |  |
| TNBC1287 | chr19 | 1203487 | 1203487 | C | T | transition | rs150335320 | *STK11* | Benign | 10.15 |  |
| TNBC1287 | chr19 | 1204889 | 1204889 | G | A | transition |  | *STK11* | Likely Benign | 3.19e-05 |  |
| TNBC1305 | chr16 | 68778858 | 68778858 | A | C | transversion | rs150045763 | *CDH1* | Likely Benign | 7.13 |  |
| TNBC1305 | chr17 | 33447304 | 33447304 | - | TTTGG | insertion | rs201291865 | *RAD51D* | Likely Benign |  |  |
| TNBC1305 | chr2 | 47630718 | 47630718 | C | G | transversion | rs535233062 | *MSH2* | Likely Benign | 2.23 |  |
| TNBC1305 | chr2 | 47631923 | 47631923 | T | A | transversion | rs550143503 | *MSH2* | Likely Benign | 8.06 |  |
| TNBC1305 | chr2 | 215675929 | 215675929 | G | C | transversion | rs143678206 | *BARD1* | Likely Benign | 3.53 |  |
| TNBC1305 | chr4 | 84407505 | 84407505 | T | C | transition |  | *ABRAXAS1* | Likely Benign |  |  |
| TNBC1316 | chr11 | 108094538 | 108094538 | G | A | transition | rs3092847 | *ATM* | Likely Benign | 15.02 |  |
| TNBC1316 | chr19 | 1205244 | 1205244 | C | T | transition |  | *STK11* | Likely Benign |  |  |
| TNBC1316 | chr7 | 152371788 | 152371788 | A | T | transversion | rs200784633 | *XRCC2* | Likely Benign |  |  |
| TNBC1316 | chr7 | 152371790 | 152371790 | T | A | transversion | rs202068983 | *XRCC2* | Likely Benign |  |  |
| TNBC1317 | chr11 | 108095034 | 108095034 | G | A | transition | rs117590923 | *ATM* | Benign | 2.25 |  |
| TNBC1317 | chr13 | 32891267 | 32891267 | C | T | transition | rs535229664 | *BRCA2* | Likely Benign | 7.26 |  |
| TNBC1317 | chr19 | 1203487 | 1203487 | C | T | transition | rs150335320 | *STK11* | Benign | 10.15 |  |
| TNBC1319 | chr10 | 89619853 | 89619853 | A | C | transversion | rs183638448 | *PTEN* | Likely Benign | 4.14 |  |
| TNBC1319 | chr2 | 215671771 | 215671771 | T | C | transition | rs113709660 | *BARD1* | Benign | 9.03 |  |
| TNBC1319 | chr2 | 215674695 | 215674695 | G | T | transversion |  | *BARD1* | Likely Benign |  |  |
| TNBC1319 | chr7 | 152373605 | 152373605 | G | A | transition | rs558961170 | *XRCC2* | Likely Benign | 6.58e-05 |  |
| TNBC1337 | chr17 | 7587687 | 7587687 | A | G | transition | rs76923748 | *TP53* | Benign | 1.46 |  |
| TNBC1337 | chr17 | 29421869 | 29421869 | A | C | transversion | rs17879128 | *NF1* | Benign | 2.28 |  |
| TNBC1337 | chr2 | 215671018 | 215671018 | T | C | transition | rs543011651 | *BARD1* | Likely Benign | 6.37e-05 |  |
| TNBC1339 | chr11 | 108101641 | 108101641 | C | G | transversion | rs762711324 | *ATM* | Likely Benign | 9.55e-05 |  |
| TNBC1339 | chr16 | 68772919 | 68772919 | T | G | transversion | rs144910517 | *CDH1* | Likely Benign | 3.04 |  |
| TNBC1344 | chr10 | 89623462 | 89623462 | G | A | transition | rs587776674 | *PTEN* | Uncertain Significance |  | Uncertain Significance |
| TNBC1344 | chr8 | 30890961 | 30890961 | C | A | transversion | rs11574155 | *WRN* | Benign | 16.28 | Uncertain Significance |
| TNBC1352 | chr11 | 94227697 | 94227697 | T | C | transition | rs1037295154 | *MRE11A* | Likely Benign | 3.19e-05 |  |
| TNBC1352 | chr15 | 91259604 | 91259604 | T | C | transition | rs534182142 | *BLM* | Likely Benign | 4.15 |  |
| TNBC1352 | chr2 | 47630607 | 47630607 | C | T | transition | rs1051833967 | *MSH2* | Likely Benign | 3.19e-05 |  |
| TNBC1355 | chr16 | 68778858 | 68778858 | A | C | transversion | rs150045763 | *CDH1* | Likely Benign | 7.13 |  |
| TNBC1355 | chr8 | 90994139 | 90994139 | C | T | transition | rs768651844 | *NBN* | Likely Benign | 3.11 |  |
| TNBC1409 | chr10 | 89624897 | 89624897 | A | G | transition |  | *PTEN* | Likely Benign |  |  |
| TNBC1409 | chr11 | 108096285 | 108096285 | C | T | transition | rs4987889 | *ATM* | Benign | 3.09 |  |
| TNBC1409 | chr16 | 68779453 | 68779453 | A | G | transition |  | *CDH1* | Likely Benign |  |  |
| TNBC1409 | chr17 | 7591538 | 7591538 | A | G | transition |  | *TP53* | Likely Benign |  |  |
| TNBC1409 | chr17 | 29421860 | 29421860 | A | T | transversion | rs144759836 | *NF1* | Likely Benign | 9.12 |  |
| TNBC1409 | chr17 | 59941842 | 59941842 | C | G | transversion |  | *BRIP1* | Likely Benign |  |  |
| TNBC1409 | chr2 | 47631993 | 47631993 | C | G | transversion | rs535395059 | *MSH2* | Likely Benign | 9.06 |  |
| TNBC1409 | chr5 | 131892836 | 131892836 | C | T | transition | rs534662635 | *RAD50* | Likely Benign | 2.23 |  |
| TNBC1425 | chr11 | 94227791 | 94227791 | A | C | transversion | rs555505826 | *MRE11A* | Likely Benign | 15.56 |  |
| TNBC1425 | chr11 | 108090479 | 108090479 | A | G | transition | rs868139795 | *ATM* | Likely Benign | 6.37e-05 |  |
| TNBC1425 | chr11 | 108095034 | 108095034 | G | A | transition | rs117590923 | *ATM* | Benign | 2.25 |  |
| TNBC1425 | chr16 | 68770715 | 68770715 | G | T | transversion | rs80089077 | *CDH1* | Benign | 5.44 |  |
| TNBC1425 | chr16 | 68778951 | 68778951 | G | A | transition | rs151267466 | *CDH1* | Likely Benign | 11.09 |  |
| TNBC1425 | chr16 | 68825347 | 68825347 | A | G | transition | rs77763492 | *CDH1* | Benign | 3.32 |  |
| TNBC1425 | chr2 | 48010991 | 48010991 | C | G | transversion |  | *MSH6* | Likely Benign | 4.79e-05 |  |
| TNBC1425 | chr7 | 6049613 | 6049613 | T | C | transition | rs77137704 | *PMS2* | Benign | 2.28 |  |
| TNBC1476 | chr11 | 108092424 | 108092424 | G | A | transition | rs190491586 | *ATM* | Likely Benign | 4.15 |  |
| TNBC1476 | chr11 | 108092647 | 108092647 | G | - | deletion | rs376953042 | *ATM* | Likely Benign | 0.35 |  |
| TNBC1476 | chr13 | 32889428 | 32889428 | G | C | transversion |  | *BRCA2* | Likely Benign |  |  |
| TNBC1476 | chr19 | 1204816 | 1204816 | C | T | transition | rs144386622 | *STK11* | Benign | 5.43 |  |
| TNBC1476 | chr19 | 1204916 | 1204916 | A | G | transition | rs3795060 | *STK11* | Benign | 2.21 |  |
| TNBC1476 | chr19 | 1210654 | 1210654 | A | G | transition | rs57782778 | *STK11* | Benign | 6.11 |  |
| TNBC1476 | chr7 | 6047765 | 6047765 | G | A | transition | rs181430858 | *PMS2* | Likely Benign | 14.21 |  |
| TNBC1487 | chr17 | 7587889 | 7587889 | A | G | transition |  | *TP53* | Likely Benign |  |  |
| TNBC1494 | chr11 | 108090462 | 108090462 | - | AAC | insertion |  | *ATM* | Likely Benign |  |  |
| TNBC1494 | chr11 | 108091177 | 108091177 | C | A | transversion | rs1442729 | *ATM* | Benign | 3.55 |  |
| TNBC1494 | chr11 | 108093002 | 108093002 | G | T | transversion | rs4987878 | *ATM* | Benign | 2.58 |  |
| TNBC1494 | chr11 | 108101838 | 108101838 | G | C | transversion | rs74706571 | *ATM* | Benign | 3.54 |  |
| TNBC1494 | chr13 | 32890122 | 32890122 | C | A | transversion |  | *BRCA2* | Likely Benign |  |  |
| TNBC1494 | chr15 | 91259752 | 91259752 | G | A | transition | rs140816542 | *BLM* | Likely Benign | 6.54 |  |
| TNBC1494 | chr16 | 23651552 | 23651552 | C | T | transition | rs189420458 | *PALB2* | Likely Benign | 3.11 |  |
| TNBC1494 | chr16 | 23652236 | 23652236 | T | C | transition | rs141315496 | *PALB2* | Likely Benign | 3.43 |  |
| TNBC1494 | chr16 | 68779243 | 68779243 | A | T | transversion | rs185033464 | *CDH1* | Likely Benign | 5.32 |  |
| TNBC1494 | chr2 | 48009137 | 48009137 | A | G | transition | rs143923556 | *MSH6* | Benign | 2.05 |  |
| TNBC1508 | chr16 | 68777254 | 68777254 | - | T | insertion | rs141699915 | *CDH1* | Likely Benign | 3.08 |  |
| TNBC1508 | chr17 | 41277354 | 41277354 | G | A | transition | rs143160357 | *BRCA1* | Likely Benign | 2.39 | Conflicting Interpretations |
| TNBC1508 | chr17 | 41278554 | 41278554 | G | T | transversion |  | *BRCA1* | Likely Benign |  |  |
| TNBC1508 | chr19 | 1207275 | 1207275 | C | G | transversion | rs1010993057 | *STK11* | Likely Benign |  |  |
| TNBC1508 | chr5 | 131893898 | 131893898 | T | C | transition | rs191149532 | *RAD50* | Likely Benign | 12.44 |  |
| TNBC1510 | chr17 | 33447370 | 33447370 | C | T | transition | rs761934757 | *RAD51D* | Likely Benign | 5.19 |  |
| TNBC1510 | chr17 | 41276360 | 41276360 | C | T | transition |  | *BRCA1* | Likely Benign |  |  |
| TNBC1510 | chr17 | 56769491 | 56769491 | G | T | transversion | rs534795003 | *RAD51C* | Likely Benign | 1.16 |  |
| TNBC1510 | chr3 | 37031336 | 37031336 | A | G | transition | rs141275247 | *MLH1* | Likely Benign | 6.38 |  |
| TNBC1510 | chr3 | 37035805 | 37035805 | A | G | transition | rs73826932 | *MLH1* | Benign | 3.09 |  |
| TNBC1510 | chr3 | 37036297 | 37036297 | - | T | insertion | rs113094640 | *MLH1* | Benign | 14.34 |  |
| TNBC1510 | chr3 | 52443962 | 52443962 | G | A | transition |  | *BAP1* | Likely Benign | 3.19e-05 | Uncertain Significance |
| TNBC1510 | chr5 | 131892948 | 131892948 | C | G | transversion |  | *RAD50* | Likely Benign |  |  |
| TNBC1511 | chr17 | 29423155 | 29423155 | C | T | transition |  | *NF1* | Likely Benign |  |  |
| TNBC1527 | chr17 | 41277944 | 41277944 | A | T | transversion | rs8176072 | *BRCA1* | Benign | 10.56 |  |
| TNBC1527 | chr2 | 47628980 | 47628980 | C | A | transversion | rs17224066 | *MSH2* | Likely Benign | 5.48 |  |
| TNBC1527 | chr4 | 84407415 | 84407415 | T | G | transversion | rs139987172 | *ABRAXAS1* | Likely Benign | 0.51 |  |
| TNBC1544 | chr19 | 1208855 | 1208855 | C | T | transition | rs113467487 | *STK11* | Benign | 3.23 |  |
| TNBC1544 | chr7 | 6048100 | 6048100 | G | T | transversion |  | *PMS2* | Likely Benign | 3.19e-05 |  |
| TNBC1544 | chr8 | 30892768 | 30892768 | A | G | transition | rs11574169 | *WRN* | Benign | 2.46 |  |
| TNBC1566 | chr11 | 108099597 | 108099597 | G | A | transition | rs779092391 | *ATM* | Likely Benign | 4.15 |  |
| TNBC1566 | chr16 | 68777254 | 68777254 | - | T | insertion | rs141699915 | *CDH1* | Likely Benign | 3.08 |  |
| TNBC1566 | chr17 | 7587847 | 7587847 | A | T | transversion | rs753643217 | *TP53* | Likely Benign | 3.11 |  |
| TNBC1566 | chr17 | 7590196 | 7590196 | A | C | transversion | rs983919925 | *TP53* | Likely Benign | 3.19e-05 |  |
| TNBC1572 | chr11 | 108095927 | 108095927 | C | G | transversion | rs926118183 | *ATM* | Likely Benign | 9.55e-05 |  |
| TNBC1572 | chr11 | 108099302 | 108099302 | C | T | transition | rs766490651 | *ATM* | Likely Benign | 9.56e-05 |  |
| TNBC1572 | chr11 | 108100454 | 108100454 | T | C | transition | rs557773874 | *ATM* | Likely Benign | 9.56e-05 |  |
| TNBC1572 | chr16 | 68780276 | 68780276 | C | T | transition | rs184562141 | *CDH1* | Likely Benign | 5.16 |  |
| TNBC1576 | chr10 | 89625582 | 89625582 | G | A | transition | rs35274268 | *PTEN* | Likely Benign | 5.09 |  |
| TNBC1576 | chr17 | 59941086 | 59941086 | A | G | transition | rs141306575 | *BRIP1* | Likely Benign | 9.55 |  |
| TNBC1576 | chr19 | 1207361 | 1207361 | G | T | transversion | rs146412237 | *STK11* | Benign | 3.59 |  |
| TNBC1576 | chr3 | 52444302 | 52444302 | G | A | transition | rs559645296 | *BAP1* | Likely Benign | 1.44 |  |
| TNBC1576 | chr3 | 52444522 | 52444522 | C | T | transition |  | *BAP1* | Likely Benign |  |  |
| TNBC1576 | chr5 | 131891608 | 131891608 | T | G | transversion | rs767032155 | *RAD50* | Likely Benign | 6.37e-05 |  |
| TNBC1576 | chr8 | 145744092 | 145744092 | C | T | transition | rs149515668 | *RECQL4* | Benign | 3.01 |  |
| TNBC1578 | chr11 | 108091168 | 108091168 | T | A | transversion | rs188903432 | *ATM* | Likely Benign | 3.09 |  |
| TNBC1578 | chr16 | 68774470 | 68774470 | - | TGGGG | insertion | rs150759824 | *CDH1* | Likely Benign |  |  |
| TNBC1578 | chr19 | 1203678 | 1203678 | T | C | transition | rs75605040 | *STK11* | Benign | 3.38 |  |
| TNBC1578 | chr19 | 1203960 | 1203964 | CTTTT | - | deletion | rs532157507 | *STK11* | Likely Benign | 9.02 |  |
| TNBC1578 | chr19 | 1208855 | 1208855 | C | T | transition | rs113467487 | *STK11* | Benign | 3.23 |  |
| TNBC1578 | chr19 | 1210513 | 1210513 | C | T | transition | rs551004937 | *STK11* | Likely Benign | 6.38e-05 |  |
| TNBC1583 | chr16 | 68781240 | 68781269 | ATGAGGCTGCAGAGGCAGCTGGGCCAGGGT | - | deletion | rs546214932 | *CDH1* | Likely Benign | 0.51 |  |
| TNBC1583 | chr16 | 68781274 | 68781274 | C | - | deletion | rs529632683 | *CDH1* | Likely Benign | 0.51 |  |
| TNBC1583 | chr17 | 29422027 | 29422027 | A | G | transition | rs1055865194 | *NF1* | Likely Benign |  |  |
| TNBC1583 | chr19 | 1206833 | 1206833 | A | C | transversion |  | *STK11* | Likely Benign |  |  |
| TNBC1583 | chr2 | 215673806 | 215673807 | CA | - | deletion | rs549956085 | *BARD1* | Likely Benign | 1.55 |  |
| TNBC1584 | chr11 | 94227093 | 94227093 | G | A | transition | rs576570834 | *MRE11A* | Likely Benign | 10.05 |  |
| TNBC1584 | chr16 | 68773561 | 68773561 | A | G | transition |  | *CDH1* | Likely Benign |  |  |
| TNBC1584 | chr17 | 56769512 | 56769512 | A | C | transversion |  | *RAD51C* | Likely Benign | 0.0 |  |
| TNBC1584 | chr17 | 59941770 | 59941770 | G | A | transition | rs776765765 | *BRIP1* | Likely Benign | 9.56e-05 |  |
| TNBC1584 | chr19 | 1203462 | 1203462 | G | - | deletion |  | *STK11* | Uncertain Significance |  |  |
| TNBC1584 | chr19 | 1209559 | 1209559 | C | T | transition | rs907807647 | *STK11* | Likely Benign |  |  |
| TNBC1584 | chr3 | 52444196 | 52444196 | A | G | transition |  | *BAP1* | Likely Benign |  |  |
| TNBC1586 | chr11 | 108091405 | 108091405 | G | T | transversion | rs185663398 | *ATM* | Likely Benign | 0.35 |  |
| TNBC1586 | chr16 | 68779175 | 68779175 | A | G | transition |  | *CDH1* | Likely Benign |  |  |
| TNBC1586 | chr17 | 29423160 | 29423160 | T | - | deletion |  | *NF1* | Benign | 12.07 |  |
| TNBC1586 | chr17 | 41277186 | 41277186 | C | A | transversion | rs1036527805 | *BRCA1* | Likely Benign | 3.2e-05 |  |
| TNBC1586 | chr17 | 41277214 | 41277214 | G | A | transition | rs894166357 | *BRCA1* | Likely Benign |  |  |
| TNBC1586 | chr3 | 37031895 | 37031895 | C | T | transition | rs987531153 | *MLH1* | Likely Benign | 10.22 |  |
| TNBC1586 | chr7 | 6051283 | 6051283 | A | G | transition | rs78848166 | *PMS2* | Benign | 2.19 |  |
| TNBC1587 | chr15 | 91260100 | 91260100 | C | T | transition | rs28364254 | *BLM* | Likely Benign | 1.33 |  |
| TNBC1591 | chr11 | 108092303 | 108092303 | C | G | transversion | rs4987874 | *ATM* | Benign | 2.16 |  |
| TNBC1591 | chr17 | 56769587 | 56769587 | G | C | transversion |  | *RAD51C* | Likely Benign |  |  |
| TNBC1591 | chr17 | 59941086 | 59941086 | A | G | transition | rs141306575 | *BRIP1* | Likely Benign | 9.55 |  |
| TNBC1591 | chr22 | 29137157 | 29137157 | - | G | insertion |  | *CHEK2* | Likely Benign |  |  |
| TNBC1591 | chr8 | 145743701 | 145743701 | G | A | transition | rs35697811 | *RECQL4* | Likely Benign | 0.21 |  |
| TNBC1592 | chr11 | 108094695 | 108094695 | G | A | transition |  | *ATM* | Likely Benign |  |  |
| TNBC1592 | chr16 | 68776189 | 68776189 | T | - | deletion | rs971880052 | *CDH1* | Likely Benign | 8.03 |  |
| TNBC1592 | chr16 | 68853448 | 68853448 | G | A | transition | rs535005673 | *CDH1* | Likely Benign | 9.57e-05 |  |
| TNBC1592 | chr19 | 1208289 | 1208289 | C | G | transversion |  | *STK11* | Likely Benign |  |  |
| TNBC1592 | chr2 | 48009721 | 48009721 | T | C | transition | rs190634663 | *MSH6* | Likely Benign | 4.47 |  |
| TNBC1592 | chr7 | 6052054 | 6052054 | C | G | transversion |  | *PMS2* | Likely Benign |  |  |
| TNBC1592 | chr8 | 145742792 | 145742792 | G | - | deletion | rs535285068 | *RECQL4* | Likely Benign |  | Conflicting Interpretations |
| TNBC1593 | chr10 | 89627667 | 89627667 | A | T | transversion | rs78815234 | *PTEN* | Benign | 0.11 |  |
| TNBC1593 | chr17 | 7587687 | 7587687 | A | G | transition | rs76923748 | *TP53* | Benign | 1.46 |  |
| TNBC1593 | chr2 | 47631828 | 47631828 | A | G | transition | rs148511604 | *MSH2* | Likely Benign | 7.14 |  |
| TNBC1632 | chr17 | 33447370 | 33447370 | C | T | transition | rs761934757 | *RAD51D* | Likely Benign | 5.19 |  |
| TNBC1632 | chr2 | 215671029 | 215671029 | C | T | transition | rs113594759 | *BARD1* | Benign | 2.41 |  |
| TNBC1642 | chr16 | 68778858 | 68778858 | A | C | transversion | rs150045763 | *CDH1* | Likely Benign | 7.13 |  |
| TNBC1642 | chr8 | 30891667 | 30891667 | A | G | transition | rs977069235 | *WRN* | Likely Benign | 3.18e-05 |  |
| TNBC1645 | chr16 | 2097850 | 2097850 | C | A | transversion |  | *NTHL1* | Likely Benign |  |  |
| TNBC1645 | chr22 | 29137157 | 29137157 | - | G | insertion |  | *CHEK2* | Likely Benign |  |  |
| TNBC1645 | chr7 | 6047765 | 6047765 | G | A | transition | rs181430858 | *PMS2* | Likely Benign | 14.21 |  |
| TNBC1647 | chr10 | 89620631 | 89620631 | A | G | transition |  | *PTEN* | Likely Benign |  |  |
| TNBC1647 | chr11 | 94227477 | 94227477 | G | A | transition | rs13447579 | *MRE11A* | Benign | 2.31 |  |
| TNBC1647 | chr11 | 108092958 | 108092958 | T | C | transition | rs940061601 | *ATM* | Likely Benign | 3.19e-05 |  |
| TNBC1647 | chr19 | 1205202 | 1205202 | T | C | transition | rs147492468 | *STK11* | Benign | 2.21 |  |
| TNBC1660 | chr11 | 108094467 | 108094467 | C | A | transversion | rs137901691 | *ATM* | Likely Benign | 4.47 |  |
| TNBC1660 | chr16 | 68777239 | 68777239 | T | C | transition |  | *CDH1* | Likely Benign | 5.51 |  |
| TNBC1660 | chr8 | 145743701 | 145743701 | G | A | transition | rs35697811 | *RECQL4* | Likely Benign | 0.21 |  |
| TNBC1686 | chr16 | 68779091 | 68779091 | G | A | transition | rs187965214 | *CDH1* | Likely Benign | 2.08 |  |
| TNBC1686 | chr17 | 59941086 | 59941086 | A | G | transition | rs141306575 | *BRIP1* | Likely Benign | 9.55 |  |
| TNBC1686 | chr7 | 152371844 | 152371844 | A | - | deletion |  | *XRCC2* | Likely Benign | 4.37e-05 |  |
| TNBC1693 | chr11 | 108096101 | 108096101 | T | C | transition |  | *ATM* | Likely Benign |  |  |
| TNBC1693 | chr7 | 6051396 | 6051396 | A | T | transversion | rs548615948 | *PMS2* | Likely Benign | 3.18e-05 |  |
| TNBC1700 | chr16 | 2098525 | 2098525 | C | A | transversion |  | *NTHL1* | Likely Benign |  |  |
| TNBC1700 | chr19 | 1203354 | 1203354 | G | A | transition | rs772745766 | *STK11* | Likely Benign | 6.54 |  |
| TNBC1700 | chr22 | 29137157 | 29137157 | - | G | insertion |  | *CHEK2* | Likely Benign |  |  |
| TNBC1700 | chr3 | 37036292 | 37036302 | TTTTTATTTTT | - | deletion | rs527871568 | *MLH1* | Likely Benign | 7.28 |  |
| TNBC1728 | chr11 | 108091673 | 108091673 | G | A | transition | rs947752483 | *ATM* | Likely Benign |  |  |
| TNBC1728 | chr11 | 108093035 | 108093035 | G | T | transversion | rs143148829 | *ATM* | Benign | 10.37 |  |
| TNBC1728 | chr11 | 108101388 | 108101388 | C | T | transition | rs151076414 | *ATM* | Benign | 11.16 |  |
| TNBC1728 | chr2 | 47629718 | 47629718 | G | T | transversion | rs17224080 | *MSH2* | Likely Benign | 4.44 |  |
| TNBC1743 | chr10 | 89624667 | 89624667 | A | G | transition | rs145409477 | *PTEN* | Likely Benign | 11.41 |  |
| TNBC1743 | chr15 | 91261377 | 91261377 | G | A | transition | rs2380157 | *BLM* | Likely Benign | 2.07 |  |
| TNBC1743 | chr16 | 23652636 | 23652636 | C | G | transversion | rs138200248 | *PALB2* | Benign | 16.31 | Conflicting Interpretations |
| TNBC1743 | chr3 | 37036546 | 37036546 | C | T | transition |  | *MLH1* | Likely Benign |  |  |
| TNBC1743 | chr7 | 152373847 | 152373847 | A | T | transversion | rs3218376 | *XRCC2* | Likely Benign | 3.15 |  |
| TNBC1761 | chr17 | 59942100 | 59942100 | T | C | transition |  | *BRIP1* | Likely Benign |  |  |
| TNBC1761 | chr8 | 145744092 | 145744092 | C | T | transition | rs149515668 | *RECQL4* | Benign | 3.01 |  |
| TNBC1773 | chr15 | 91260100 | 91260100 | C | T | transition | rs28364254 | *BLM* | Likely Benign | 1.33 |  |
| TNBC1773 | chr17 | 59941770 | 59941770 | G | A | transition | rs776765765 | *BRIP1* | Likely Benign | 9.56e-05 |  |
| TNBC1773 | chr8 | 90995426 | 90995426 | C | T | transition | rs118026302 | *NBN* | Likely Benign | 3.12 |  |
| TNBC1787 | chr7 | 6049400 | 6049400 | G | A | transition | rs191847792 | *PMS2* | Benign | 0.25 |  |
| TNBC1795 | chr15 | 91260735 | 91260735 | G | T | transversion | rs28364256 | *BLM* | Likely Benign | 4.44 |  |
| TNBC1795 | chr16 | 68777317 | 68777317 | A | G | transition | rs114740986 | *CDH1* | Benign | 4.45 |  |
| TNBC1798 | chr11 | 108101646 | 108101646 | C | T | transition |  | *ATM* | Likely Benign |  |  |
| TNBC1798 | chr2 | 48011904 | 48011904 | T | C | transition | rs547694242 | *MSH6* | Likely Benign | 3.2e-05 |  |
| TNBC1812 | chr10 | 89624636 | 89624636 | A | G | transition | rs187088207 | *PTEN* | Likely Benign | 16.27 |  |
| TNBC1812 | chr15 | 91260735 | 91260735 | G | T | transversion | rs28364256 | *BLM* | Likely Benign | 4.44 |  |
| TNBC1812 | chr16 | 68775552 | 68775552 | A | - | deletion |  | *CDH1* | Likely Benign | 4.39 |  |
| TNBC1812 | chr17 | 29423149 | 29423149 | - | C | insertion | rs367998687 | *NF1* | Likely Benign |  |  |
| TNBC1812 | chr2 | 215675939 | 215675939 | G | C | transversion |  | *BARD1* | Likely Benign |  |  |
| TNBC1812 | chr7 | 6052220 | 6052220 | A | T | transversion | rs148315304 | *PMS2* | Benign | 13.38 |  |
| TNBC1812 | chr7 | 152371795 | 152371795 | T | - | deletion | rs369577931 | *XRCC2* | Likely Benign |  |  |
| TNBC1818 | chr19 | 1208055 | 1208055 | C | G | transversion | rs34973804 | *STK11* | Benign | 4.47 |  |
| TNBC1818 | chr8 | 145743743 | 145743743 | G | A | transition | rs956224680 | *RECQL4* | Likely Benign | 9.02 |  |
| TNBC1822 | chr10 | 89619853 | 89619853 | A | C | transversion | rs183638448 | *PTEN* | Likely Benign | 4.14 |  |
| TNBC1822 | chr10 | 89625125 | 89625125 | C | T | transition | rs575550829 | *PTEN* | Likely Benign | 3.11 |  |
| TNBC1822 | chr10 | 89625341 | 89625341 | T | C | transition | rs148541239 | *PTEN* | Likely Benign | 2.42 |  |
| TNBC1822 | chr10 | 89625796 | 89625796 | A | G | transition | rs542841208 | *PTEN* | Likely Benign | 2.46 |  |
| TNBC1822 | chr10 | 89626256 | 89626256 | - | A | insertion | rs527649472 | *PTEN* | Uncertain Significance |  |  |
| TNBC1822 | chr10 | 89626746 | 89626746 | A | G | transition | rs147923172 | *PTEN* | Likely Benign | 3.11 |  |
| TNBC1822 | chr10 | 89627045 | 89627046 | TA | - | deletion | rs547801894 | *PTEN* | Likely Benign | 3.11 |  |
| TNBC1822 | chr10 | 89627093 | 89627093 | G | A | transition | rs150045420 | *PTEN* | Likely Benign | 2.43 |  |
| TNBC1822 | chr10 | 89627138 | 89627138 | G | A | transition | rs185905407 | *PTEN* | Likely Benign | 0.16 |  |
| TNBC1822 | chr10 | 89627465 | 89627465 | T | G | transversion | rs144936555 | *PTEN* | Likely Benign | 3.11 |  |
| TNBC1822 | chr10 | 89628162 | 89628162 | C | G | transversion | rs140379294 | *PTEN* | Benign | 3.12 |  |
| TNBC1822 | chr10 | 89628175 | 89628175 | T | - | deletion | rs879790506 | *PTEN* | Benign | 4.23 |  |
| TNBC1822 | chr10 | 89628252 | 89628252 | A | C | transversion | rs554930875 | *PTEN* | Likely Benign | 3.11 |  |
| TNBC1822 | chr10 | 89628451 | 89628451 | T | G | transversion | rs148069374 | *PTEN* | Likely Benign | 3.11 |  |
| TNBC1822 | chr10 | 89628644 | 89628644 | A | T | transversion | rs138206799 | *PTEN* | Likely Benign | 3.11 |  |
| TNBC1822 | chr10 | 89628738 | 89628738 | T | C | transition | rs187306018 | *PTEN* | Likely Benign | 3.11 |  |
| TNBC1822 | chr10 | 89629021 | 89629021 | C | T | transition | rs565305240 | *PTEN* | Likely Benign | 3.38 |  |
| TNBC1822 | chr10 | 89629085 | 89629085 | C | T | transition | rs147232299 | *PTEN* | Likely Benign | 3.09 |  |
| TNBC1822 | chr16 | 68770771 | 68770771 | C | G | transversion | rs117397448 | *CDH1* | Likely Benign | 10.28 |  |
| TNBC1825 | chr2 | 215673484 | 215673484 | T | A | transversion | rs575390104 | *BARD1* | Likely Benign | 6.37e-05 |  |
| TNBC1831 | chr11 | 108097721 | 108097721 | A | G | transition | rs79211913 | *ATM* | Benign | 9.18 |  |
| TNBC1831 | chr13 | 32889643 | 32889643 | G | A | transition |  | *BRCA2* | Likely Benign |  |  |
| TNBC1831 | chr16 | 23651442 | 23651442 | G | A | transition | rs1049272104 | *PALB2* | Likely Benign | 6.37e-05 |  |
| TNBC1831 | chr16 | 68779243 | 68779243 | A | T | transversion | rs185033464 | *CDH1* | Likely Benign | 5.32 |  |
| TNBC1831 | chr2 | 215674695 | 215674695 | G | T | transversion |  | *BARD1* | Likely Benign |  |  |
| TNBC1831 | chr3 | 37030229 | 37030229 | T | C | transition | rs4678557 | *MLH1* | Benign | 9.06 |  |
| TNBC1842 | chr11 | 94227240 | 94227240 | T | A | transversion | rs919031219 | *MRE11A* | Likely Benign | 3.43 |  |
| TNBC1842 | chr11 | 94227241 | 94227241 | G | T | transversion | rs932960780 | *MRE11A* | Likely Benign | 3.43 |  |
| TNBC1842 | chr16 | 68770771 | 68770771 | C | G | transversion | rs117397448 | *CDH1* | Likely Benign | 10.28 |  |
| TNBC1842 | chr17 | 41277944 | 41277944 | A | T | transversion | rs8176072 | *BRCA1* | Benign | 10.56 |  |
| TNBC1842 | chr17 | 59941037 | 59941037 | A | G | transition | rs545287870 | *BRIP1* | Likely Benign |  |  |
| TNBC1855 | chr5 | 131893782 | 131893782 | G | A | transition | rs78524273 | *RAD50* | Benign | 3.17 |  |
| TNBC1869 | chr10 | 89622735 | 89622736 | AG | - | deletion | rs556751243 | *PTEN* | Likely Benign | 3.02 |  |
| TNBC1869 | chr15 | 91261911 | 91261911 | A | G | transition | rs115535358 | *BLM* | Benign | 8.36 |  |
| TNBC1869 | chr17 | 33445435 | 33445435 | G | T | transversion | rs181672547 | *RAD51D* | Benign | 1.42 |  |
| TNBC1869 | chr17 | 41277361 | 41277361 | C | T | transition | rs544857747 | *BRCA1* | Likely Benign |  |  |
| TNBC1869 | chr7 | 6048775 | 6048775 | G | A | transition |  | *PMS2* | Likely Benign |  |  |
| TNBC1869 | chr7 | 6052220 | 6052220 | A | T | transversion | rs148315304 | *PMS2* | Benign | 13.38 |  |
| TNBC1894 | chr19 | 1202963 | 1202963 | G | A | transition | rs563682217 | *STK11* | Likely Benign | 6.39e-05 |  |
| TNBC1894 | chr2 | 48009290 | 48009290 | T | G | transversion |  | *MSH6* | Likely Benign |  |  |
| TNBC1894 | chr2 | 215673830 | 215673830 | G | C | transversion | rs546617980 | *BARD1* | Benign | 5.19 |  |
| TNBC1915 | chr10 | 89623633 | 89623633 | A | T | transversion |  | *PTEN* | Likely Benign | 6.67e-05 |  |
| TNBC1915 | chr11 | 108099782 | 108099784 | TGA | - | deletion | rs761958021 | *ATM* | Likely Benign | 3.19e-05 |  |
| TNBC1915 | chr16 | 68779447 | 68779447 | A | T | transversion |  | *CDH1* | Benign | 2.46 |  |
| TNBC1915 | chr17 | 29421869 | 29421869 | A | C | transversion | rs17879128 | *NF1* | Benign | 2.28 |  |
| TNBC1931 | chr16 | 68778858 | 68778858 | A | C | transversion | rs150045763 | *CDH1* | Likely Benign | 7.13 |  |
| TNBC1961 | chr2 | 215673806 | 215673807 | CA | - | deletion | rs549956085 | *BARD1* | Likely Benign | 1.55 |  |
| TNBC1983 | chr5 | 131893782 | 131893782 | G | A | transition | rs78524273 | *RAD50* | Benign | 3.17 |  |
| TNBC1983 | chr7 | 6051625 | 6051625 | T | C | transition | rs1038279773 | *PMS2* | Likely Benign |  |  |
| TNBC1983 | chr7 | 152372119 | 152372119 | G | C | transversion |  | *XRCC2* | Likely Benign |  |  |
| TNBC1983 | chr8 | 30891346 | 30891346 | A | T | transversion | rs1015768505 | *WRN* | Likely Benign |  |  |
| TNBC1983 | chr8 | 145742792 | 145742792 | G | - | deletion | rs535285068 | *RECQL4* | Likely Benign |  | Conflicting Interpretations |
| TNBC1991 | chr16 | 23652769 | 23652769 | G | C | transversion | rs552824227 | *PALB2* | Likely Benign | 3.33 |  |
| TNBC1991 | chr16 | 68772366 | 68772366 | G | A | transition |  | *CDH1* | Likely Benign |  |  |
| TNBC1991 | chr17 | 29423789 | 29423789 | G | C | transversion | rs150326083 | *NF1* | Benign | 0.56 |  |
| TNBC1991 | chr22 | 29137163 | 29137163 | T | C | transition | rs17885379 | *CHEK2* | Benign | 2.35 |  |
| TNBC1991 | chr7 | 6052727 | 6052727 | C | T | transition | rs146016964 | *PMS2* | Likely Benign | 14.53 |  |
| TNBC2006 | chr11 | 94227477 | 94227477 | G | A | transition | rs13447579 | *MRE11A* | Benign | 2.31 |  |
| TNBC2006 | chr19 | 1207361 | 1207361 | G | T | transversion | rs146412237 | *STK11* | Benign | 3.59 |  |
| TNBC2006 | chr22 | 29137163 | 29137163 | T | C | transition | rs17885379 | *CHEK2* | Benign | 2.35 |  |
| TNBC2006 | chr4 | 84406068 | 84406068 | C | G | transversion | rs560610909 | *ABRAXAS1* | Likely Benign | 5.19 |  |
| TNBC2010 | chr11 | 108099374 | 108099374 | G | A | transition | rs79866170 | *ATM* | Benign | 2.22 |  |
| TNBC2010 | chr17 | 41277944 | 41277944 | A | T | transversion | rs8176072 | *BRCA1* | Benign | 10.56 |  |
| TNBC2010 | chr19 | 1203363 | 1203363 | G | A | transition | rs192063406 | *STK11* | Likely Benign | 0.15 |  |
| TNBC2010 | chr19 | 1205196 | 1205196 | C | - | deletion | rs538652196 | *STK11* | Likely Benign | 15.18 |  |
| TNBC2010 | chr19 | 1205197 | 1205197 | C | T | transition | rs559847307 | *STK11* | Likely Benign | 15.17 |  |
| TNBC2011 | chr16 | 68774939 | 68774939 | G | A | transition | rs181460012 | *CDH1* | Likely Benign | 2.11 |  |
| TNBC2011 | chr2 | 215671836 | 215671836 | A | G | transition | rs148183199 | *BARD1* | Benign | 5.16 |  |
| TNBC2011 | chr3 | 37030124 | 37030124 | G | C | transversion | rs942593958 | *MLH1* | Likely Benign |  |  |
| TNBC2011 | chr3 | 37031564 | 37031564 | G | C | transversion | rs1039262311 | *MLH1* | Likely Benign |  |  |
| TNBC2024 | chr17 | 41277384 | 41277384 | G | A | transition | rs548750620 | *BRCA1* | Likely Benign | 3.18e-05 | Uncertain Significance |
| TNBC2024 | chr7 | 152372074 | 152372074 | G | C | transversion |  | *XRCC2* | Likely Benign |  |  |
| TNBC2036 | chr10 | 89626726 | 89626726 | G | C | transversion |  | *PTEN* | Likely Benign |  |  |
| TNBC2036 | chr16 | 68775552 | 68775552 | A | - | deletion |  | *CDH1* | Likely Benign | 4.39 |  |
| TNBC2036 | chr19 | 1208463 | 1208463 | C | T | transition | rs531224898 | *STK11* | Likely Benign | 3.3e-05 |  |
| TNBC2044 | chr11 | 108100446 | 108100446 | A | T | transversion | rs757798564 | *ATM* | Likely Benign | 3.19e-05 |  |
| TNBC2044 | chr15 | 91261911 | 91261911 | A | G | transition | rs115535358 | *BLM* | Benign | 8.36 |  |
| TNBC2044 | chr16 | 68772919 | 68772919 | T | G | transversion | rs144910517 | *CDH1* | Likely Benign | 3.04 |  |
| TNBC2045 | chr11 | 108097700 | 108097700 | A | T | transversion | rs183666845 | *ATM* | Likely Benign | 2.56 |  |
| TNBC2045 | chr17 | 59941532 | 59941536 | AAAGA | - | deletion | rs528178795 | *BRIP1* | Benign | 2.37 |  |
| TNBC2045 | chr19 | 1205202 | 1205202 | T | C | transition | rs147492468 | *STK11* | Benign | 2.21 |  |
| TNBC2046 | chr16 | 68779453 | 68779453 | A | G | transition |  | *CDH1* | Likely Benign |  |  |
| TNBC2055 | chr19 | 1203811 | 1203811 | G | T | transversion | rs137923191 | *STK11* | Likely Benign | 0.29 |  |
| TNBC2055 | chr19 | 1204770 | 1204770 | T | G | transversion | rs190616081 | *STK11* | Likely Benign | 10.17 |  |
| TNBC2055 | chr19 | 1205196 | 1205196 | C | - | deletion | rs538652196 | *STK11* | Likely Benign | 15.18 |  |
| TNBC2055 | chr19 | 1205197 | 1205197 | C | T | transition | rs559847307 | *STK11* | Likely Benign | 15.17 |  |
| TNBC2055 | chr2 | 47631993 | 47631993 | C | G | transversion | rs535395059 | *MSH2* | Likely Benign | 9.06 |  |
| TNBC2055 | chr7 | 6048345 | 6048345 | G | T | transversion |  | *PMS2* | Likely Benign | 3.2e-05 |  |
| TNBC2066 | chr11 | 94226398 | 94226398 | T | A | transversion | rs183829476 | *MRE11A* | Likely Benign | 4.28 | Other |
| TNBC2066 | chr11 | 94226459 | 94226459 | G | A | transition | rs397509350 | *MRE11A* | Likely Benign | 4.28 | Other |
| TNBC2066 | chr17 | 41277944 | 41277944 | A | T | transversion | rs8176072 | *BRCA1* | Benign | 10.56 |  |
| TNBC2066 | chr2 | 215671836 | 215671836 | A | G | transition | rs148183199 | *BARD1* | Benign | 5.16 |  |
| TNBC2068 | chr11 | 108096921 | 108096921 | A | G | transition | rs567780894 | *ATM* | Likely Benign | 5.51 |  |
| TNBC2068 | chr7 | 6051283 | 6051283 | A | G | transition | rs78848166 | *PMS2* | Benign | 2.19 |  |
| TNBC2078 | chr11 | 108097836 | 108097836 | G | C | transversion |  | *ATM* | Likely Benign |  |  |
| TNBC2078 | chr19 | 1207286 | 1207286 | C | T | transition | rs77762532 | *STK11* | Benign | 4.56 |  |
| TNBC2078 | chr2 | 47631828 | 47631828 | A | G | transition | rs148511604 | *MSH2* | Likely Benign | 7.14 |  |
| TNBC2078 | chr22 | 29137095 | 29137095 | G | A | transition | rs140000715 | *CHEK2* | Likely Benign | 15.25 |  |
| TNBC2078 | chr7 | 6048097 | 6048097 | A | G | transition |  | *PMS2* | Likely Benign |  |  |
| TNBC2084 | chr11 | 94227791 | 94227791 | A | C | transversion | rs555505826 | *MRE11A* | Likely Benign | 15.56 |  |
| TNBC2084 | chr11 | 108097622 | 108097622 | T | C | transition | rs919178595 | *ATM* | Likely Benign |  |  |
| TNBC2084 | chr16 | 68774556 | 68774556 | T | C | transition | rs184494693 | *CDH1* | Likely Benign | 6.37e-05 |  |
| TNBC2085 | chr19 | 1208368 | 1208368 | G | A | transition | rs143918180 | *STK11* | Benign | 13.41 |  |
| TNBC2085 | chr7 | 152373916 | 152373916 | - | A | insertion |  | *XRCC2* | Likely Benign | 0.0 |  |
| TNBC2096 | chr10 | 89621061 | 89621061 | C | T | transition | rs7901097 | *PTEN* | Benign | 0.27 |  |
| TNBC2096 | chr10 | 89624763 | 89624763 | C | T | transition | rs34308750 | *PTEN* | Likely Benign | 5.57 |  |
| TNBC2096 | chr10 | 89627371 | 89627371 | T | G | transversion | rs549920513 | *PTEN* | Likely Benign | 7.26 |  |
| TNBC2096 | chr11 | 108099374 | 108099374 | G | A | transition | rs79866170 | *ATM* | Benign | 2.22 |  |
| TNBC2096 | chr16 | 68775551 | 68775551 | C | A | transversion | rs79846694 | *CDH1* | Likely Benign | 4.24 |  |
| TNBC2096 | chr16 | 68777595 | 68777595 | G | C | transversion | rs116607960 | *CDH1* | Benign | 16.06 |  |
| TNBC2096 | chr17 | 29421163 | 29421163 | G | A | transition |  | *NF1* | Likely Benign |  |  |
| TNBC2096 | chr19 | 1203678 | 1203678 | T | C | transition | rs75605040 | *STK11* | Benign | 3.38 |  |
| TNBC2096 | chr19 | 1208855 | 1208855 | C | T | transition | rs113467487 | *STK11* | Benign | 3.23 |  |
| TNBC2096 | chr19 | 1210881 | 1210881 | G | A | transition | rs576420418 | *STK11* | Benign | 4.48 |  |
| TNBC2096 | chr2 | 47629319 | 47629319 | A | C | transversion | rs17217674 | *MSH2* | Benign | 2.53 |  |
| TNBC2096 | chr2 | 47631288 | 47631289 | TG | - | deletion | rs17217737 | *MSH2* | Benign | 3.05 |  |
| TNBC2096 | chr2 | 47631791 | 47631791 | T | C | transition | rs72884632 | *MSH2* | Benign | 3.01 |  |
| TNBC2096 | chr3 | 37032356 | 37032356 | A | G | transition | rs62244262 | *MLH1* | Likely Benign | 7.26 |  |
| TNBC2096 | chr4 | 84405171 | 84405171 | C | T | transition | rs76009998 | *ABRAXAS1* | Benign | 3.58 |  |
| TNBC2096 | chr4 | 84405172 | 84405172 | C | T | transition | rs77408148 | *ABRAXAS1* | Benign | 4.51 |  |
| TNBC2096 | chr4 | 84405406 | 84405406 | T | C | transition | rs79273060 | *ABRAXAS1* | Benign | 3.59 |  |
| TNBC2096 | chr4 | 84406969 | 84406969 | G | C | transversion | rs80290794 | *ABRAXAS1* | Benign | 4.56 |  |
| TNBC2096 | chr7 | 152373709 | 152373709 | G | C | transversion |  | *XRCC2* | Likely Benign |  |  |
| TNBC2107 | chr10 | 89623678 | 89623678 | - | GCG | insertion | rs34413673 | *PTEN* | Uncertain Significance |  |  |
| TNBC2107 | chr17 | 56769512 | 56769512 | A | C | transversion |  | *RAD51C* | Likely Benign | 0.0 |  |
| TNBC2107 | chr3 | 37030229 | 37030229 | T | C | transition | rs4678557 | *MLH1* | Benign | 9.06 |  |
| TNBC2107 | chr5 | 131894223 | 131894223 | C | T | transition | rs186664261 | *RAD50* | Likely Benign | 7.31 |  |
| TNBC2107 | chr7 | 152371846 | 152371847 | TA | - | deletion |  | *XRCC2* | Likely Benign | 4.79e-05 |  |
| TNBC2108 | chr15 | 91260952 | 91260952 | A | G | transition |  | *BLM* | Likely Benign |  |  |
| TNBC2108 | chr16 | 68775552 | 68775552 | A | - | deletion |  | *CDH1* | Likely Benign | 4.39 |  |
| TNBC2108 | chr17 | 56769512 | 56769512 | A | C | transversion |  | *RAD51C* | Likely Benign | 0.0 |  |
| TNBC2108 | chr2 | 47628980 | 47628980 | C | A | transversion | rs17224066 | *MSH2* | Likely Benign | 5.48 |  |
| TNBC2114 | chr2 | 47631091 | 47631091 | G | C | transversion | rs142758375 | *MSH2* | Likely Benign | 1.05 |  |
| TNBC2114 | chr2 | 47631828 | 47631828 | A | G | transition | rs148511604 | *MSH2* | Likely Benign | 7.14 |  |
| TNBC2120 | chr11 | 94227423 | 94227423 | G | A | transition | rs13447580 | *MRE11A* | Benign | 2.24 |  |
| TNBC2120 | chr2 | 48010355 | 48010355 | G | T | transversion | rs199913053 | *MSH6* | Likely Benign | 4.47 | Conflicting Interpretations |
| TNBC2120 | chr22 | 29138032 | 29138032 | C | T | transition | rs745351020 | *CHEK2* | Likely Benign | 3.19e-05 |  |
| TNBC2120 | chr8 | 145743938 | 145743938 | C | T | transition | rs895229899 | *RECQL4* | Likely Benign | 3.18e-05 |  |
| TNBC2133 | chr11 | 108099597 | 108099597 | G | A | transition | rs779092391 | *ATM* | Likely Benign | 4.15 |  |
| TNBC2133 | chr17 | 29423149 | 29423149 | - | C | insertion | rs367998687 | *NF1* | Likely Benign |  |  |
| TNBC2133 | chr17 | 59941657 | 59941657 | C | T | transition |  | *BRIP1* | Likely Benign |  |  |
| TNBC2133 | chr19 | 1204770 | 1204770 | T | G | transversion | rs190616081 | *STK11* | Likely Benign | 10.17 |  |
| TNBC2133 | chr19 | 1205196 | 1205196 | C | - | deletion | rs538652196 | *STK11* | Likely Benign | 15.18 |  |
| TNBC2133 | chr19 | 1205197 | 1205197 | C | T | transition | rs559847307 | *STK11* | Likely Benign | 15.17 |  |
| TNBC2133 | chr2 | 48010987 | 48010987 | A | G | transition |  | *MSH6* | Likely Benign | 3.24 |  |
| TNBC2164 | chr11 | 108092303 | 108092303 | C | G | transversion | rs4987874 | *ATM* | Benign | 2.16 |  |
| TNBC2164 | chr11 | 108092724 | 108092724 | G | A | transition | rs36220458 | *ATM* | Likely Benign | 6.22 |  |
| TNBC2164 | chr16 | 68774796 | 68774796 | A | T | transversion | rs960357321 | *CDH1* | Likely Benign |  |  |
| TNBC2164 | chr16 | 68774939 | 68774939 | G | A | transition | rs181460012 | *CDH1* | Likely Benign | 2.11 |  |
| TNBC2164 | chr17 | 59939991 | 59939991 | T | C | transition | rs559026792 | *BRIP1* | Likely Benign | 2.39 |  |
| TNBC2164 | chr19 | 1203793 | 1203795 | GAG | - | deletion | rs531467701 | *STK11* | Uncertain Significance | 8.31 |  |
| TNBC2167 | chr16 | 68770771 | 68770771 | C | G | transversion | rs117397448 | *CDH1* | Likely Benign | 10.28 |  |
| TNBC2167 | chr17 | 7589927 | 7589927 | T | C | transition |  | *TP53* | Likely Benign |  |  |
| TNBC2167 | chr7 | 6049326 | 6049326 | A | T | transversion | rs747366075 | *PMS2* | Likely Benign | 3.13 |  |
| TNBC2197 | chr19 | 1208055 | 1208055 | C | G | transversion | rs34973804 | *STK11* | Benign | 4.47 |  |
| TNBC2197 | chr22 | 29138990 | 29138990 | G | A | transition |  | *CHEK2* | Likely Benign |  |  |
| TNBC2197 | chr7 | 152373385 | 152373385 | T | G | transversion | rs767350140 | *XRCC2* | Likely Benign | 3.11 |  |
| TNBC2197 | chr8 | 30890581 | 30890581 | G | A | transition |  | *WRN* | Likely Benign |  |  |
| TNBC2216 | chr11 | 108101888 | 108101888 | A | G | transition |  | *ATM* | Likely Benign |  |  |
| TNBC2216 | chr2 | 47631710 | 47631710 | C | G | transversion | rs892959277 | *MSH2* | Likely Benign | 9.57e-05 |  |
| TNBC2217 | chr16 | 68779447 | 68779447 | A | T | transversion |  | *CDH1* | Benign | 2.46 |  |
| TNBC2217 | chr17 | 29421869 | 29421869 | A | C | transversion | rs17879128 | *NF1* | Benign | 2.28 |  |
| TNBC2217 | chr19 | 1208055 | 1208055 | C | G | transversion | rs34973804 | *STK11* | Benign | 4.47 |  |
| TNBC2217 | chr2 | 47631710 | 47631710 | C | T | transition | rs892959277 | *MSH2* | Likely Benign | 6.38e-05 |  |
| TNBC2217 | chr2 | 47631828 | 47631828 | A | G | transition | rs148511604 | *MSH2* | Likely Benign | 7.14 |  |
| TNBC2239 | chr17 | 7587687 | 7587687 | A | G | transition | rs76923748 | *TP53* | Benign | 1.46 |  |
| TNBC2239 | chr17 | 41277944 | 41277944 | A | T | transversion | rs8176072 | *BRCA1* | Benign | 10.56 |  |
| TNBC2239 | chr22 | 29137163 | 29137163 | T | C | transition | rs17885379 | *CHEK2* | Benign | 2.35 |  |
| TNBC2239 | chr5 | 131892606 | 131892606 | G | T | transversion |  | *RAD50* | Likely Benign |  |  |
| TNBC2239 | chr8 | 90995606 | 90995606 | G | A | transition |  | *NBN* | Likely Benign |  |  |
| TNBC2245 | chr11 | 94227879 | 94227879 | A | C | transversion | rs13447578 | *MRE11A* | Benign | 5.05 |  |
| TNBC2245 | chr11 | 108095390 | 108095390 | C | T | transition |  | *ATM* | Likely Benign |  |  |
| TNBC2245 | chr11 | 108097960 | 108097960 | G | A | transition |  | *ATM* | Likely Benign |  |  |
| TNBC2245 | chr17 | 59941086 | 59941086 | A | G | transition | rs141306575 | *BRIP1* | Likely Benign | 9.55 |  |
| TNBC2245 | chr2 | 215675466 | 215675466 | A | C | transversion | rs112488105 | *BARD1* | Benign | 1.59 |  |
| TNBC2256 | chr16 | 68770650 | 68770650 | C | G | transversion | rs34475632 | *CDH1* | Likely Benign | 3.44 |  |
| TNBC2256 | chr16 | 68771143 | 68771143 | C | T | transition | rs34500817 | *CDH1* | Likely Benign | 0.23 |  |
| TNBC2256 | chr16 | 68776010 | 68776010 | G | A | transition | rs118106511 | *CDH1* | Likely Benign | 0.24 |  |
| TNBC2256 | chr16 | 68776911 | 68776911 | G | A | transition | rs139471434 | *CDH1* | Likely Benign | 3.49 |  |
| TNBC2256 | chr16 | 68777234 | 68777234 | C | A | transversion | rs562648086 | *CDH1* | Benign | 5.31 |  |
| TNBC2256 | chr16 | 68779508 | 68779508 | - | TG | insertion |  | *CDH1* | Likely Benign |  |  |
| TNBC2256 | chr16 | 68780276 | 68780276 | C | T | transition | rs184562141 | *CDH1* | Likely Benign | 5.16 |  |
| TNBC2256 | chr16 | 68780725 | 68780725 | G | C | transversion | rs144506739 | *CDH1* | Likely Benign | 0.23 |  |
| TNBC2256 | chr19 | 1207989 | 1207989 | G | A | transition | rs571455034 | *STK11* | Benign | 0.36 |  |
| TNBC2256 | chr2 | 215675466 | 215675466 | A | C | transversion | rs112488105 | *BARD1* | Benign | 1.59 |  |
| TNBC2256 | chr22 | 29137095 | 29137095 | G | A | transition | rs140000715 | *CHEK2* | Likely Benign | 15.25 |  |
| TNBC2266 | chr10 | 89625582 | 89625582 | G | A | transition | rs35274268 | *PTEN* | Likely Benign | 5.09 |  |
| TNBC2266 | chr17 | 41278542 | 41278542 | G | A | transition | rs868099576 | *BRCA1* | Likely Benign | 1.58 |  |
| TNBC2266 | chr19 | 1209293 | 1209293 | G | T | transversion |  | *STK11* | Likely Benign |  |  |
| TNBC2266 | chr5 | 131893984 | 131893984 | T | A | transversion | rs73257758 | *RAD50* | Benign | 2.25 |  |
| TNBC2279 | chr11 | 94227423 | 94227423 | G | A | transition | rs13447580 | *MRE11A* | Benign | 2.24 |  |
| TNBC2279 | chr7 | 152373847 | 152373847 | A | T | transversion | rs3218376 | *XRCC2* | Likely Benign | 3.15 |  |
| TNBC2279 | chr8 | 30892199 | 30892199 | A | G | transition | rs761633705 | *WRN* | Likely Benign | 9.56e-05 |  |
| TNBC2279 | chr8 | 90996027 | 90996027 | G | C | transversion |  | *NBN* | Likely Benign |  |  |
| TNBC2316 | chr19 | 1210325 | 1210325 | C | T | transition | rs61612016 | *STK11* | Benign | 10.05 |  |
| TNBC2316 | chr2 | 48010922 | 48010922 | G | C | transversion |  | *MSH6* | Likely Benign |  |  |
| TNBC2316 | chr2 | 215675466 | 215675466 | A | C | transversion | rs112488105 | *BARD1* | Benign | 1.59 |  |
| TNBC2345 | chr10 | 89626194 | 89626194 | G | T | transversion |  | *PTEN* | Likely Benign |  |  |
| TNBC2345 | chr11 | 108094538 | 108094538 | G | A | transition | rs3092847 | *ATM* | Likely Benign | 15.02 |  |
| TNBC2345 | chr15 | 91259989 | 91259992 | AAGT | - | deletion | rs142264462 | *BLM* | Benign | 3.56 |  |
| TNBC2345 | chr16 | 68770776 | 68770776 | G | T | transversion | rs192929184 | *CDH1* | Likely Benign | 3.05 |  |
| TNBC2345 | chr17 | 29423150 | 29423150 | C | - | deletion |  | *NF1* | Benign | 8.06 |  |
| TNBC2345 | chr19 | 1210384 | 1210384 | G | A | transition | rs181578411 | *STK11* | Benign | 2.36 |  |
| TNBC2345 | chr2 | 215675341 | 215675341 | - | T | insertion | rs888838784 | *BARD1* | Likely Benign |  |  |
| TNBC2360 | chr19 | 1207361 | 1207361 | G | T | transversion | rs146412237 | *STK11* | Benign | 3.59 |  |
| TNBC2360 | chr19 | 1208802 | 1208803 | AG | - | deletion |  | *STK11* | Likely Benign | 3.86e-05 |  |
| TNBC2360 | chr19 | 1208803 | 1208803 | - | TTT | insertion |  | *STK11* | Likely Benign |  |  |
| TNBC2360 | chr7 | 6049191 | 6049191 | C | T | transition | rs151162174 | *PMS2* | Benign | 4.56 |  |
| TNBC2374 | chr11 | 108094538 | 108094538 | G | A | transition | rs3092847 | *ATM* | Likely Benign | 15.02 |  |
| TNBC2374 | chr16 | 68847112 | 68847112 | A | C | transversion | rs894169196 | *CDH1* | Likely Benign |  |  |
| TNBC2374 | chr17 | 33446907 | 33446911 | CCTGG | - | deletion | rs1057517577 | *RAD51D* | Likely Benign | 6.4e-05 | Uncertain Significance |
| TNBC2374 | chr2 | 47631828 | 47631828 | A | G | transition | rs148511604 | *MSH2* | Likely Benign | 7.14 |  |
| TNBC2383 | chr11 | 94228127 | 94228127 | T | G | transversion | rs36225559 | *MRE11A* | Likely Benign | 15.59 |  |
| TNBC2383 | chr13 | 32891267 | 32891267 | C | T | transition | rs535229664 | *BRCA2* | Likely Benign | 7.26 |  |
| TNBC2383 | chr16 | 2096713 | 2096713 | C | G | transversion | rs562415277 | *NTHL1* | Likely Benign | 4.18 |  |
| TNBC2383 | chr16 | 2099006 | 2099006 | C | T | transition |  | *NTHL1* | Likely Benign |  |  |
| TNBC2383 | chr17 | 29421860 | 29421860 | A | T | transversion | rs144759836 | *NF1* | Likely Benign | 9.12 |  |
| TNBC2383 | chr2 | 47629319 | 47629319 | A | C | transversion | rs17217674 | *MSH2* | Benign | 2.53 |  |
| TNBC2383 | chr2 | 47629433 | 47629433 | C | T | transition |  | *MSH2* | Likely Benign | 3.19e-05 |  |
| TNBC2383 | chr7 | 6051283 | 6051283 | A | G | transition | rs78848166 | *PMS2* | Benign | 2.19 |  |
| TNBC2487 | chr11 | 108093974 | 108093974 | A | G | transition | rs1800066 | *ATM* | Likely Benign | 0.59 |  |
| TNBC2487 | chr11 | 108100222 | 108100222 | - | T | insertion | rs201519743 | *ATM* | Likely Benign |  |  |
| TNBC2487 | chr19 | 1209910 | 1209910 | C | T | transition | rs149200995 | *STK11* | Benign | 14.12 |  |
| TNBC2487 | chr7 | 6049620 | 6049620 | - | TATATATATATG | insertion |  | *PMS2* | Likely Benign |  |  |
| TNBC2487 | chr8 | 145743994 | 145743994 | G | A | transition | rs1019042369 | *RECQL4* | Likely Benign | 9.55e-05 |  |
| TNBC2499 | chr19 | 1209910 | 1209910 | C | T | transition | rs149200995 | *STK11* | Benign | 14.12 |  |
| TNBC2539 | chr11 | 94227423 | 94227423 | G | A | transition | rs13447580 | *MRE11A* | Benign | 2.24 |  |
| TNBC2539 | chr11 | 108092303 | 108092303 | C | G | transversion | rs4987874 | *ATM* | Benign | 2.16 |  |
| TNBC2539 | chr15 | 91260100 | 91260100 | C | T | transition | rs28364254 | *BLM* | Likely Benign | 1.33 |  |
| TNBC2539 | chr17 | 29421860 | 29421860 | A | T | transversion | rs144759836 | *NF1* | Likely Benign | 9.12 |  |
| TNBC2539 | chr2 | 215675466 | 215675466 | A | C | transversion | rs112488105 | *BARD1* | Benign | 1.59 |  |
| TNBC2546 | chr11 | 94226398 | 94226398 | T | A | transversion | rs183829476 | *MRE11A* | Likely Benign | 4.28 | Other |
| TNBC2546 | chr11 | 94226459 | 94226459 | G | A | transition | rs397509350 | *MRE11A* | Likely Benign | 4.28 | Other |
| TNBC2546 | chr16 | 68775552 | 68775552 | A | - | deletion |  | *CDH1* | Likely Benign | 4.39 |  |
| TNBC2546 | chr17 | 59942093 | 59942093 | G | C | transversion | rs1031835230 | *BRIP1* | Likely Benign | 3.18e-05 |  |
| TNBC2546 | chr19 | 1207989 | 1207989 | G | A | transition | rs571455034 | *STK11* | Benign | 0.36 |  |
| TNBC2546 | chr7 | 6049191 | 6049191 | C | T | transition | rs151162174 | *PMS2* | Benign | 4.56 |  |
| TNBC2587 | chr10 | 89622735 | 89622736 | AG | - | deletion | rs556751243 | *PTEN* | Likely Benign | 3.02 |  |
| TNBC2587 | chr11 | 94227423 | 94227423 | G | A | transition | rs13447580 | *MRE11A* | Benign | 2.24 |  |
| TNBC2587 | chr2 | 48010111 | 48010111 | C | G | transversion | rs551212403 | *MSH6* | Likely Benign | 0.33 |  |
| TNBC2587 | chr3 | 37036292 | 37036302 | TTTTTATTTTT | - | deletion | rs527871568 | *MLH1* | Likely Benign | 7.28 |  |
| TNBC2587 | chr4 | 84407194 | 84407194 | A | C | transversion |  | *ABRAXAS1* | Likely Benign |  |  |
| TNBC2597 | chr11 | 108096366 | 108096366 | A | G | transition | rs1045834231 | *ATM* | Likely Benign |  |  |
| TNBC2597 | chr22 | 29137095 | 29137095 | G | A | transition | rs140000715 | *CHEK2* | Likely Benign | 15.25 |  |

**Supplementary Table 1:** This table describes the 635 rare variants identified in the 144 patients with TNBC, the chromosomal location, the reference and alternative allele, the type of variants, the public databases of pathogenicity interpretation, and the frequency in the population.
