# Supplementary material for: Exploring the promoter regions of cancer predisposition genes in patients with triple-negative breast cancer reveals the presence of rare germline variants
Source: Oncologist. 2025 May 8;30(5):oyaf052. doi: 10.1093/oncolo/oyaf052 (PMC12060721; doi:10.1093/oncolo/oyaf052)

**Supplementary Figure 1.** The oncoprint figure of genetic alterations identified on patients’ samples. Columns represent individual, blood samples and are sorted by amount of alterations. Each gene affected by alterations has been reported and sorted by frequency (indicated on the left). The different colors represent the different alterations, as described in the legend on the figure. The bar plots at the top and right of the oncoprint indicate the count of events found respectively in each sample and in each gene. In the lower part of the oncoprint, familiarity and age at diagnosis are reported.


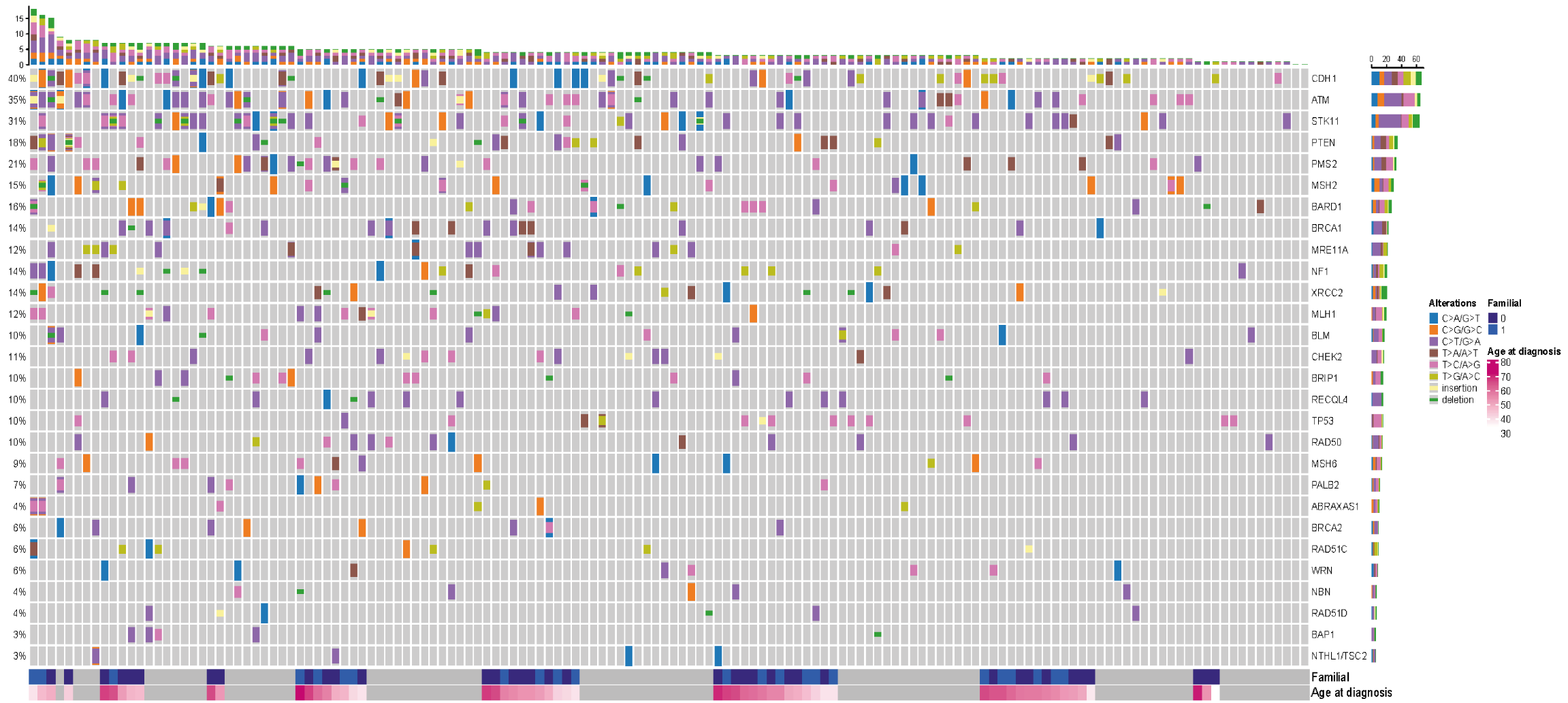

Supplement: oyaf052_suppl_Supplementary_Figures_1 [file oyaf052_suppl_supplementary_figures_1.docx]
